# Microscopic and Proteomic Analysis of Dissected Developing Barley Endosperm Layers Reveals the Starchy Endosperm as Prominent Storage Tissue for ER-Derived Hordeins Alongside the Accumulation of Barley Protein Disulfide Isomerase (HvPDIL1-1)

**DOI:** 10.3389/fpls.2018.01248

**Published:** 2018-09-10

**Authors:** Valentin Roustan, Pierre-Jean Roustan, Marieluise Weidinger, Siegfried Reipert, Eszter Kapusi, Azita Shabrangy, Eva Stoger, Wolfram Weckwerth, Verena Ibl

**Affiliations:** ^1^Department of Ecogenomics and Systems Biology, University of Vienna, Vienna, Austria; ^2^Cell Imaging and Ultrastructure Research, University of Vienna, Vienna, Austria; ^3^Department for Applied Genetics and Cell Biology, University of Natural Resources and Life Sciences, Vienna, Austria; ^4^Vienna Metabolomics Center, University of Vienna, Vienna, Austria

**Keywords:** shotgun proteomics, confocal microscopy, seed storage proteins, barley, PDIL1-1, grain development, tissue-specific, protein bodies

## Abstract

Barley (*Hordeum vulgare*) is one of the major food sources for humans and forage sources for animal livestock. The average grain protein content (GPC) of barley ranges between 8 and 12%. Barley hordeins (i.e., prolamins) account for more than 50% of GPC in mature seeds and are important for both grain and flour quality. Barley endosperm is structured into three distinct cell layers: the starchy endosperm, which acts essentially as storage tissue for starch; the subaleurone, which is characterized by a high accumulation of seed storage proteins (SSPs); and the aleurone, which has a prominent role during seed germination. Prolamins accumulate in distinct, ER-derived protein bodies (PBs) and their trafficking route is spatio-temporally regulated. The protein disulfide isomerase (PDI) has been shown to be involved in PB formation. Here, we unravel the spatio-temporal proteome regulation in barley aleurone, subaleurone, and starchy endosperm for the optimization of end-product quality in barley. We used laser microdissection (LMD) for subsequent nanoLC-MS/MS proteomic analyses in two experiments: in Experiment One, we investigated the proteomes of dissected barley endosperm layers at 12 and at ≥20 days after pollination (DAP). We found a set of 10 proteins that were present in all tissues at both time points. Among these proteins, the relative protein abundance of D-hordein, B3-hordein and HvPDIL1-1 significantly increased in starchy endosperm between 12 and ≥20 DAP, identifying the starchy endosperm as putative major storage tissue. In Experiment Two, we specifically compared the starchy endosperm proteome at 6, 12, and ≥20 DAP. Whereas the relative protein abundance of D-hordein and B3-hordein increased between 6 and ≥20 DAP, HvPDIL1-1 increased between 6 and 12 DAP, but remained constant at ≥20 DAP. Microscopic observations showed that these relative protein abundance alterations were accompanied by additional localization of hordeins at the periphery of starch granules and a partial re-localization of HvPDIL1-1 from PBs to the periphery of starch granules. Our data indicate a spatio-temporal regulation of hordeins and HvPDIL1-1. These results are discussed in relation to the putative role of HvPDIL1-1 in end-product quality in barley.

## Introduction

Barley is a cereal crop mainly used for food, animal feed and beverage production. Barley is the fourth-most crop in terms of food production after maize, wheat and rice (FAOSTAT 2016; www.fao.org). Barley grain texture and/or grain protein content (GPC) is an important quality determinant for end uses such as malt production, animal feed as well as food (Cai et al., 2013). The average GPC typically ranges between 8 and 12% (Shewry and Halford, 2002; Cai et al., 2013).

Cereal seed proteins can be classified according to their solubility into four groups: albumins, globulins, prolamins, and glutelin. Albumins and globulins are soluble in water and dilute salt solutions, respectively. Prolamins are soluble in aqueous alcohol and glutelins are the remaining proteins not extracted by either solvent (Shewry and Tatham, 1990). Cereal seed proteins are also classified into two groups according to their biological function: seed storage proteins (SSPs) and non-storage proteins (Shewry and Tatham, 1990). SSPs can belong either to prolamins, glutelins, or globulins family. In barley, prolamins are termed hordeins, and account for more than 50% of the total protein amount in mature seeds (Shewry and Tatham, 1990; Shewry, 1999). Together with other SSPs, hordeins are important for both grain and flour quality (Psota et al., 2007). Hordeins are classified into three groups: sulfur rich (B + γ), sulfur poor (C), and high molecular weight prolamins (D). B and C hordeins together account for over 95% of the total hordein content (Rahman et al., 1984).

The major storage tissue of cereal grains is the endosperm. Barley caryopsis endosperm is composed of different cell layers with different spatio-temporal physiological and molecular mechanisms (Olsen, 2001, 2004). The aleurone layer regulates the germination process by secreting necessary enzymes to break down stored reserves. The outer layer of the endosperm, the subaleurone layer, stores high levels of SSP whereas the inner starchy endosperm layer accumulates both starch and SSPs (Young and Gallie, 2000; Moore et al., 2016). Prolamins accumulate in ER-derived protein bodies (PBs) and are most abundant in the starchy endosperm (Shewry and Halford, 2002). In barley, hordeins show a differential distribution pattern where subaleurone cells are enriched in S-rich and S-poor B- and C-hordeins while D-hordeins are only present in the inner part of the starchy endosperm (Tosi et al., 2011).

During development, SSP transport routes depend on the cereal species, endosperm tissue layer and developmental timepoint (Ibl and Stoger, 2012; Arcalis et al., 2014; Ibl et al., 2014; Zheng and Wang, 2014). In general, SSPs are synthesized on the rough ER and translocated into the ER lumen. In barley, PBs are taken up by the protein storage vacuoles and finally released in a spatio-temporal manner in the developing endosperm (Ibl et al., 2014). In rice endosperm, protein oxidative folding proceeds and the formation of disulfide bonds triggers protein transport into PBs (Kawagoe et al., 2005).

Thus, disulfide transfer pathways play a critical role in the formation of PBs in cereal endosperm (reviewed in Onda, 2013). The introduction of disulfide bonds requires an interplay of a disulfide generating enzyme (e.g., ER oxidoreductase 1) and a disulfide carrier protein (e.g., protein disulfide isomerase). Protein disulfide isomerases (PDIs) are folding catalysts that interact with nascent polypeptides to aid the formation of proper disulfide bonds during protein folding (reviewed in Freedman et al., 1994; Onda, 2013). The genomes of *Arabidopsis thaliana*, rice (*Oryza sativa*), maize (*Zea mays*), soybean (*Glycine max*), and wheat (*Triticum aestivum*) encode diverse PDIs that can be classified into 10 subgroups (Houston et al., 2005; Kimura et al., 2015), although all PDIs contain two thioredoxin (TRX)-fold redox-active domains. These domains consist of a pair of Cys residues involved in thiol-disulfide exchange reactions. Such reactions necessarily involve ER oxidoreductase 1 (ERO1); thus, the redox state of disulfide bonds in SSPs and, consequently, of PBs changes during cereal seed development (Onda, 2013). PDIs are multidomain proteins and thus vary widely in their functions. Nevertheless, PDI members of soybean, maize and rice have been suggested to be involved in oxidative folding of SSPs (Onda, 2013). The rice *esp2* mutant, which lacks PDIL1;1, showed an inhibition of the formation of native disulfide bonds resulting in the anomalous interaction of proglutelin and prolamin polypeptides in newly formed ER PBs (Takemoto et al., 2002; Onda et al., 2009; Satoh-Cruz et al., 2010).

A recent shotgun proteomic study on mature barley seeds enabled more complete characterization of the barley seed proteome (Mahalingam, 2017). Our recent shotgun proteomic analyses unraveled the spatio-temporal relative protein abundance and subcellular localization of hordoindolines across development in barley endosperm (Shabrangy et al., 2018). However, a survey of the spatio-temporal distribution of proteins, especially SSPs and ER-related proteins during barley grain filling is still missing.

The main objectives of this study were the following: first, we wanted to describe the proteomes of dissected developing barley endosperm tissues. For proteomic analyses we prepared cryosections from aleurone, subaleurone, and starchy endosperm by laser microdissection (LMD) of barley grains harvested at 12 and ≥20 DAP. Protein extracts were analyzed by nanoLC-MS/MS methods. Qualitative and quantitative proteome profiling of the different cell layers revealed tissue-specific changes in relative protein abundances and identified the starchy endosperm as the main protein storage tissue. Hordeins and HvPDIL1-1 were identified as highly abundant proteins that were most expressed in the starchy endosperm at 12 and ≥20 DAP. In Experiment Two, six stage-specific clusters were identified; D-hordein, B3-hordein and HvPDIL1-1 clustered in group Two and Three, respectively, where the relative protein abundance of all proteins continuously increased between 6 and ≥20 DAP or remained stable to ≥20 DAP. Along with the protein relative abundance alterations of D-hordein, B3-hordein and HvPDIL1-1, microscopic studies showed a subcellular re-localization of hordeins and HvPDIL1-1 indicating a fusion of PBs and ER structures with the protein matrix at the periphery of the starch granule. Possible roles of HvPDIL1-1 in starchy endosperm development are discussed in terms of cereal food end-product quality and molecular farming.

## Materials and methods

### Plasmids, plant material, and growth conditions

Barley (*Hordeum vulgare L*.) wild-type variety Golden Promise (GP) (kindly provided by IPK Gatersleben) and its transgenic derivative spGFP-PDIL1;1 were cultivated as previously described (Ibl et al., 2014; Shabrangy et al., 2018). OsTIP3::spGFP-PDIL1;1 plasmid was a gift of Yasushi Kawagoe and is described in Onda et al. (2009). Transgenic plants were generated by particle bombardment following (Ibl et al., 2014; Hilscher et al., 2016; Kapusi et al., 2017). Transgenic lines were screened by PCR, and Western blot was used to confirm a stable spGFP-PDIL1;1 transgenic barley line (Figure S1) as following: Developing seeds for the spGFP-PDIL1;1 transgenic line and GP as negative control were harvested. Fresh grain (100 mg) were homogenized in 2 ml reducing agent (25 mM Tris-HCl pH 7.8, 1.6% SDS, 100 mM DTT) using pestle and mortar on ice. The homogenate was centrifuged at 6,800 g for 15 min at 4°C and the supernatant was transferred into a fresh microtube. SDS-PAGE sample buffer [20 μl containing 250 mM Tris–HCl (pH 6.8), 10% (w/v) SDS, 0.5% (w/v) bromophenol blue, and 50% (v/v) glycerol] was added to 80 μl of each sample and subsequently boiled for 5 min in microtubes, cooled at room temperature. They were loaded on 5% stacking polyacrylamide gel and fractionated in 15% resolving gel for 2 h at a constant current of 25 mA under denaturing conditions using a Bio-Rad mini-gel electrophoresis unit. The starting voltage was 52 V and the final voltage 124 V. The electrode buffer consisted of 25 mM Tris-base (pH 8.8), 200 mM glycine, and 0.1% (w/v) SDS. After electrophoresis, the gel was presoaked in the blotting buffer [48 mM Tris-base (pH 8.3), 39 mM glycine, 20% (v/v) methanol], together with 3 mm Whatman filter paper and nitrocellulose membrane for 30 min. Transfer of proteins from the gel to nitrocellulose membrane was done using the Bio-Rad semi-dry transblotter. The electroblotting was done at a constant voltage of 18 V for 30 min. Following the transfer, the nitrocellulose membrane was blocked in 5% (w/v) non-fat “carnation” powdered milk (1 h) prepared in phosphate buffered with saline Tween 20 (PBST) (pH 7.4). The immunoblot was incubated in 1:5,000 dilution of anti-GFP rabbit antiserum (Abcam) prepared in PBST buffer for 2 h at room temperature, followed by washing 3 times in PBST, 5 min each. The second antibody was anti-rabbit IgG–alkaline phosphatase conjugate prepared fresh at 1:5,000 dilution and incubated for 1 h. The immunostaining was performed using Bio-Rad ready-to-use reagents.

### Tissue preparation for laser microdissection (LMD)

Following (Shabrangy et al., 2018), three replicate caryopses were harvested at 6, 12, and ≥20 DAP and the tissues prepared for LMD.

### Database searches and bioinformatic analyses of PDI cDNAs and proteins

Specific primers were designed for *HvPDIL1-1* using Primer-Blast (https://www.ncbi.nlm.nih.gov/tools/primer-blast/) resulting in small, specific PCR products (Table S1). The Arabidopsis PDI protein sequences at IPK Gatersleben homepage (http://webblast.ipk-gatersleben.de/barley_ibsc/) were used for TBLASTN to search for full-length cDNA in *H. vulgare*. Each accession number of full-length cDNA was taken to search for the corresponding protein in UniProt. The alignment of all *PDI* cDNAs as well as of all PDI protein sequences was performed by MEGA7.0.21 (Figures S2, S3; Kumar et al., 2016). The phylogenetic tree also was constructed by MEGA7.0.21 (Kumar et al., 2016) using a neighbor-joining statistical method including 1,000 bootstrap replications. The alignments were visualized using GeneDoc (Nicholas and Nicholas, 1997). The conserved percentage was marked as black = 100%, dark gray = 80%.

### RT-qPCR analysis

RT-qPCR analysis of *HvPDIL1-1* was performed according to the MIQE guidelines (Bustin et al., 2009) using previously extracted and qualitatively screened RNA isolated from the whole seed as well as from LMD sections from 12 to ≥20 DAP (Shabrangy et al., 2018). cDNA was synthesized as recently described (Shabrangy et al., 2018). Additionally, RNA was isolated from starchy endosperm from 6 DAP. Concentration was measured at 260 nm using a UV spectrophotometer (NanoDrop Technologies, Thermo Fisher Scientific, Waltham, MA, USA) and was between 36 and 41 ng/μl. RNA integrity was assessed by a microfluidic capillary gel electrophoresis applying the ExperionTM system (ExperionTM RNA HighSens Analysis Kit, Bio-Rad Laboratories, Hercules, CA, USA) with Experion software 3.2P. The quality of RNA index (QRI) was between possibly acceptable quality and acceptable quality. At least three biological replicates were used and three pipetting replicates were performed for RT-qPCR. For the normalization studies of *HvPDIL1-1* transcripts, we used the following reference genes as described in Shabrangy et al. (2018): ARF (ADP-ribosylation factor), FBPA (fructose-bisphosphate aldolase), and SAM (S-adenosyl-L-methionine) for whole seed (WS); GAP (glyceraldehyde-3-phosphate dehydrogenase), GRP (glycine-rich protein), and UBI (ubiquitin gene) for LMD at 12 DAP; HSP90 (heat shock protein 90), ACPIII (acyl carrier protein III), and ARF for LMD at ≥20 DAP; HSP70 (Heat shock 70 kD protein), HSP90, GRP, ELF (elongation factor 1-alpha), UBI, and FBPA for AL (12 and ≥20 DAP); SAM, GRP, HSP70, ARF, HSP90, FBPA, ELF, and UBI for SA (12 and ≥20 DAP); and ELF, FBPA, and UBI for SE (12 and ≥20 DAP). Normalization was calculated as described (Vandesompele et al., 2002; Shabrangy et al., 2018). For statistical analyses we performed a Student's *t-*Test [(two-tailed distribution, two-sample unequal variance (heteroscedastic)] by the software Microsoft Excel.

### Microscopy

Fluorescence microscopy of developing barley grains was performed using polyclonal rabbit anti-AtPDIL1-1 antibody (dilution 1:50) and polyclonal rabbit anti-gliadin antibody (Sigma-Aldrich; dilution 1:100) as described in Shabrangy et al. (2018).

For microscopic fluorescence quantification, at least three barley seeds were harvested at 6 and ≥20 DAP, sectioned and washed with tap water. The sections were incubated with ER-Tracker™ Green BODIPY™ FL Glibenclamide (Thermo Fisher Scientific) following (Ibl et al., 2014). The sections were observed by Nikon Eclipse Ni, and 5-10 aleurone, subaleurone, and starchy endosperm areas were quantified by ImageJ (Schneider et al., 2012). For statistical analyses, *t-*tests were used and calculated by Microsoft Excel.

Images for confocal microscopy were captured using the Leica SP5 CLSM with filter settings for autofluorescence (excitation wavelength 405 nm, emission wavelength 410–480 nm), GFP (excitation 488 nm, emission 500–530 nm), ER-Tracker™ Red (excitation 561 nm, emission 571–623 nm), and BES-H_2_O_2_-Ac (excitation wavelength 488 nm, emission wavelength 500–535 nm). A time series (4 s interval, 2 min movie) for spGFP-PDIL1;1 labeled ER was taken (Movie S1). Images were processed using Leica confocal software version 3.5, ImageJ and Adobe Photoshop 12.0.4.

For the diameter quantification of the anti-AtPDIL1-1- and anti-gliadin-positive PBs, at least two slides containing three sections each were analyzed and the diameter of 100 PBs was measured each time by the “straight tool” (μm) of the ImageJ software in subaleurone and starchy endosperm at 6, 12, and ≥20 DAP of anti AtPDIL1-1-positive PBs (Figures S4A,B) and of anti-gliadin-positive PBs in starchy endosperm at 6, 12, and ≥20 DAP, respectively (Figure S5). For statistical analyses we performed a Student's *t-*Test [(two-tailed distribution, two-sample unequal variance (homoscedastic)] by the software Microsoft Excel.

### Sample preparation and nano-HPLC coupled MS/MS measurement

Sample preparations were performed as previously described (Shabrangy et al., 2018). Briefly, proteins were extracted from dissected cells collected by LMD from barley grains harvested at 6, 12, and ≥20 DAP, in three independent replicates. Extraction was performed following a phenol-phase separation protocol. Subsequently, proteins were re-suspended in a urea buffer and protein concentration was measured with a Bradford Assay prior to trypsin digestion. Following overnight digestion, the peptides were desalted with C18 solid phase extraction (SPE) (Agilent Technologies, Santa Clara, CA, USA). After desalting, the corresponding eluate was dried in a vacuum concentrator.

Similarly, samples were analyzed as previously described by nano-HPLC coupled to MS/MS methods (Shabrangy et al., 2018). Peptides were resolved at a protein concentration equivalent of 0.1 μg/μl in 5% (v/v) ACN, 0.1% (v/v) formic acid. A total of 0.5 μg of the mixture was separated on an EASY-Spray PepMap RSLC 75 μm × 50 cm column (Thermo Fisher Scientific Inc., Waltham, MA, USA). Peptides were eluted using a 150 min linear gradient from 2 to 40% of mobile phase B (mobile phase A: 0.1% [v/v] formic acid in water; mobile phase B: 0.1% [v/v] formic acid in 90% [v/v] acetonitrile) with 300 nL/min flow rate generated with an UltiMate 3000 RSLCnano system. Peptides were measured with an LTQ-Orbitrap Elite (Thermo Fisher Scientific Inc., Waltham, MA, USA), using same mass analyzer settings as in Shabrangy et al. (2018).

### Data processing and protein identification

Raw files were processed with MaxQuant 1.5 (http://www.maxquant.org) and the Andromeda search algorithm (Cox and Mann, 2008; Cox et al., 2011; Tyanova et al., 2015) on the barley UniProt database with the same settings as Shabrangy et al. (2018). Label-free quantification was done at the peptide level with at least two peptides per proteins. PTXQC was used to assess data quality. The final dataset represented proteins quantified in three biological replicates in at least one group of samples in both Experiment One and Two (Table S2). Statistical analysis was completed with Perseus 1.5 software (Bielow et al., 2016; Tyanova, 2016) including a one-way ANOVA for the Experiment Two. In both datasets, each protein of interest was subjected to a Student's *t-*Test [(two-tailed distribution, two-sample unequal variance (heteroscedastic)] within the software Microsoft Excel. For Experiment Two, cluster analysis was performed with fuzzy-c means algorithm implemented in GProX (Rigbolt et al., 2011), and protein-protein interaction (PPI) networks were implemented by STRING with default parameters (Franceschini et al., 2013). The mass spectrometry proteomic data have been deposited to the ProteomeXchange Consortium (Deutsch et al., 2017) via the PRIDE (Vizcano et al., 2016) partner repository with the dataset identifier PXD009708, PXD009710, and PXD009722.

## Results

### Proteomes of dissected developing barley endosperm layers

The three layers of barley endosperm, the aleurone, subaleurone, and starchy endosperm are differentially regulated. Nevertheless, these three cell layers act together during grain development to ensure sufficient amino acid and carbohydrate storage to fuel the embryo during germination (Domínguez and Cejudo, 2014). In this context, the distribution of SSPs is tissue specific. The subaleurone stores high amounts of SSPs and the starchy endosperm both starch and SSPs (Moore et al., 2016).

Until now, manual dissections of aleurone and the remaining endosperm have been analyzed with 2D-gel–based mass spectrometry (Finnie et al., 2002, 2004, 2011; Hynek et al., 2006). Kaspar et al. (2010) coupled LMD with LC-MS/MS analysis to unravel the proteome composition of nucellar projections and endosperm transfer cells. Recently, we performed an LMD approach to unravel the spatio-temporal gene expression, relative protein abundance and subcellular localization of hordoindolines across barley endosperm development (Shabrangy et al., 2018). Here, we precisely dissect and describe the spatio-temporal composition of the proteome of aleurone, subaleurone, and starchy endosperm.

**Figure 1:**
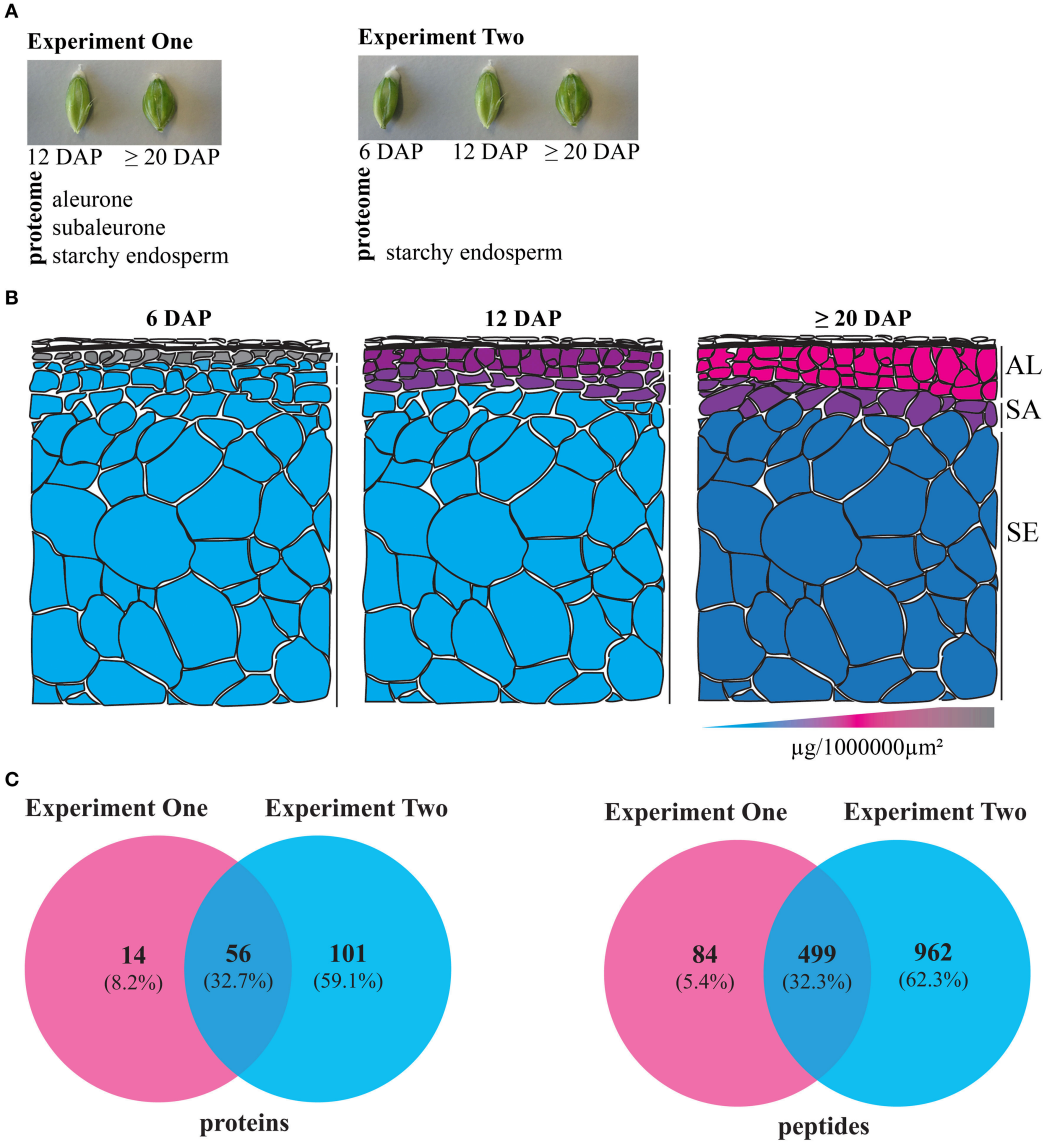
Summary of the two LMD experiments to study the proteomes of dissected developing barley endosperm layers. **(A)** Overview of the proteomic analyses of dissected developing barley endosperm layers: in Experiment One, we performed proteomic analyses on dissected aleurone, subaleurone, and starchy endosperm at 12 and ≥20 DAP. In Experiment Two, we performed proteomics analyses only on dissected starchy endosperm at 6, 12, and ≥20 DAP. **(B)** Schematic overview representing the alterations of the relative protein abundance profiles in aleurone (AL), subaleurone (SA), and starchy endosperm (SE) between 6, 12, and ≥20 DAP. Note the 3-color-schema: blue represents the lowest value, pink represent the median value, and gray represents the highest value. **(C)** Venn diagrams illustrate the quantified proteins and peptides in Experiments One and Two.

Two experiments were conducted: In Experiment One we analyzed the spatio-temporal distribution of the proteome at 12 and ≥20 DAP, while in Experiment Two we focused on the starchy endosperm development at 6, 12, and ≥20 DAP (Figure 1A). For the first time, the distribution of protein content across the aleurone, subaleurone, and starchy endosperm could be quantified (Table S2). Interestingly, the relative protein abundance over the surface was the lowest for the starchy endosperm (Figure 1B). We observed only little variation in the relative protein abundance within the tissues during the grain filling: statistical analyses showed significant differences in the relative protein abundance per 1,000,000 μm^2^ only for the subaleurone layer between 6 and 12 DAP (Figure 1B). At 6 DAP, the relative protein abundance was significantly different between starchy endosperm and aleurone and between subaleurone and aleurone (Figure 1B). At 12 DAP, a significant difference of the relative protein abundance could be detected between subaleurone and starchy endosperm (Figure 1B). Our proteomic approach identified and quantified 583 and 1,461 peptides in Experiments One and Two, respectively (Figure 1C, Table S2 sheets B,D). Based on a quantification parameter of at least two peptides per protein at each time point in each tissue, 246 and 504 proteins were quantified in Experiments One and Two, respectively. Proteins identified and quantified in three biological replicates in at least one group of samples were incorporated into a final dataset. Consequently, Experiments One and Two comprised 70 and 157 proteins, respectively (Figure 1C, Table S2 sheets A,C). Identified proteins were functionally annotated using the Mercator tool (Lohse et al., 2014). More than 80% of peptides and proteins detected in the spatio-temporal study was also detected in the starchy endosperm (Experiment Two).

**Figure 2:**
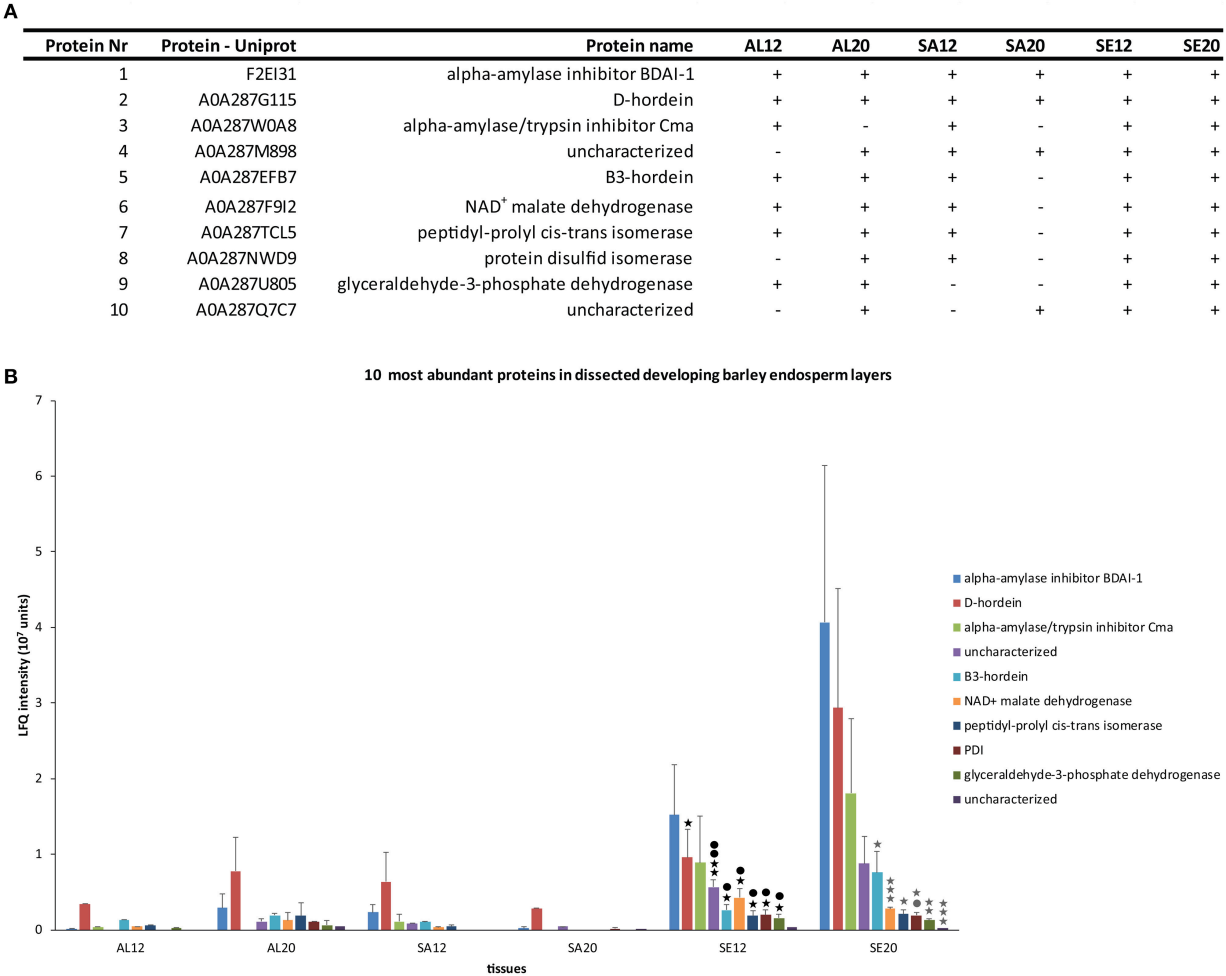
Characterization of 10 proteins identified in at least four different groups of samples. **(A)** Table of identified proteins including their protein UniProt number, protein name and the LMD samples where the proteins were identified (+). **(B)** LFQ intensity of ten identified proteins in aleurone 12 DAP (AL12), and 20 DAP (AL20), subaleurone 12 DAP (SA12) and ≥ 20 DAP (SA20) and starchy endosperm at 12 DAP (SE12) and ≥ 20 DAP (SE20). LFQ intensities of proteins were averaged over three replications. Bars represent standard deviation. For statistical analyses we performed a Student's *t*-test (*n* = 3). Significant *p-*values indicate as following: asterisk black = significant change between AL12-SE12; dot black = significant change between SA12-SE12; gray asterisk = significant change between SA20-SE20; gray dot = significant change between AL20-SE20. One asterisk/dot = *p* < 0.05; two asterisks/dots = *p* < 0.01 and three asterisks/dots = *p* < 0.001.

To restrict our analysis to the most informative results, we focused on proteins which could be measured in at least four different groups of samples. This filtering resulted in a set of 10 proteins (Figure 2A): alpha-amylase inhibitor BDAI-1, D-hordein, alpha-amylase/trypsin inhibitor Cma, B3-hordein, NAD^+^ malate dehydrogenase, peptidyl-prolyl cis-trans isomerase, PDI, glyceraldehyde-3-phosphate dehydrogenase, and two uncharacterized barley proteins. D-hordein, B3-hordein, NAD^+^ malate dehydrogenase, peptidyl-prolyl cis-trans isomerase, PDI, glyceraldehyde-3-phosphate dehydrogenase, and both uncharacterized barley proteins accumulated significantly more in the starchy endosperm at 12 and ≥20 DAP than in the other tissues (Figure 2B). Additionally, a group of proteins that were more abundant in the aleurone than in the starchy endosperm at ≥20 DAP could be detected (Table S2 sheet A). Among them, ricin-like proteins involved in seed defense mechanisms (Domashevskiy and Goss, 2015), protein synthesis–associated and degradation-associated proteins could be identified. Interestingly, a DNA repair protein was found in the aleurone but not in the starchy endosperm. These results indicate that the starchy endosperm is the major storage layer of these identified proteins. Additionally, we identified hordeins and PDI within the subset of the 10 most expressed proteins that are most abundant in the starchy endosperm at 12 and ≥20 DAP.

### Spatio-temporal relative protein abundance quantification and localization changes of hordeins during barley endosperm development

**Figure 3:**
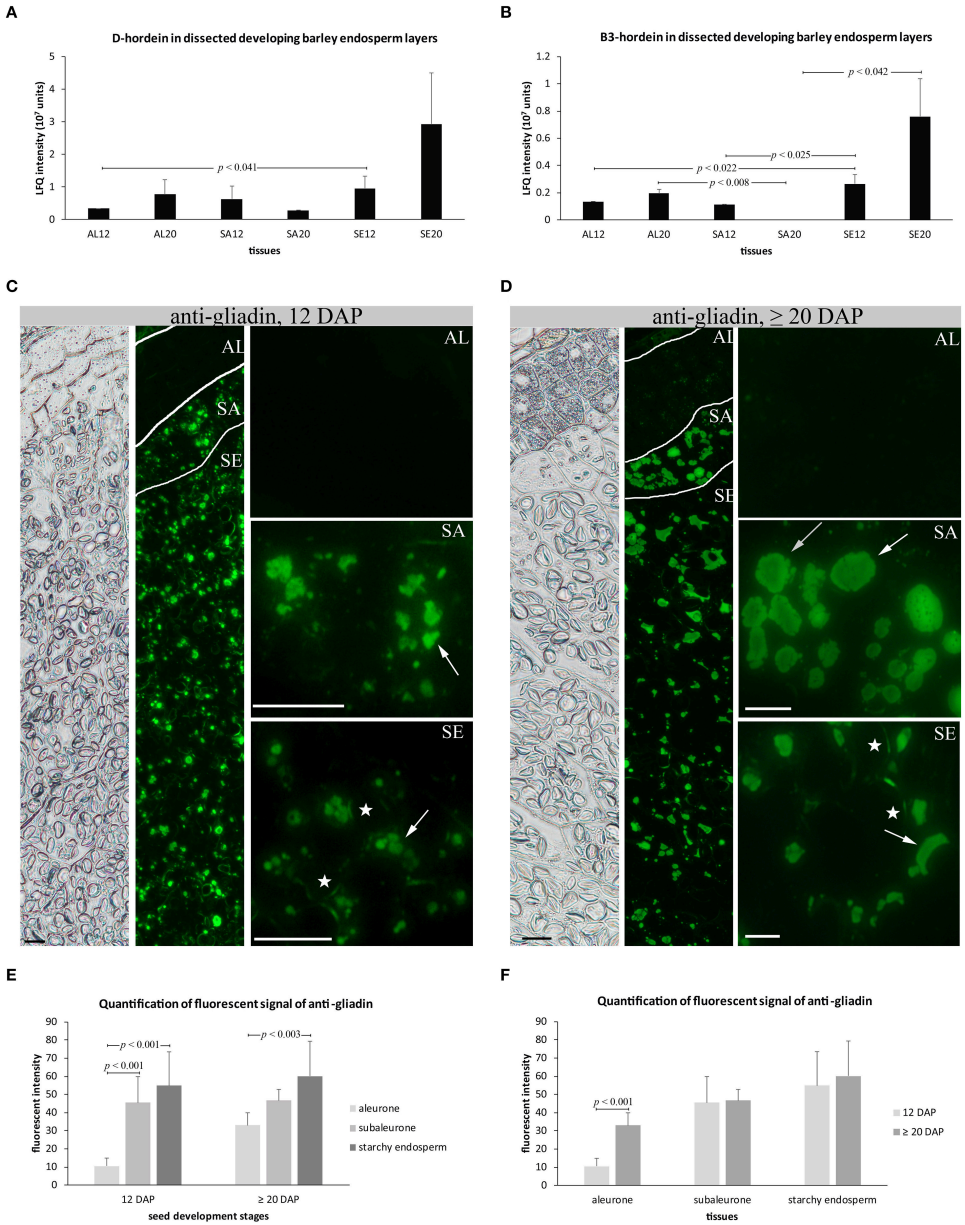
Quantification and subcellular distribution of hordeins at 12 and ≥20 DAP of endosperm development. **(A,B)** LFQ intensity of D-hordein and B3-hordein of aleurone 12 DAP (AL12) and 20 DAP (AL20), subaleurone 12 DAP (SA12), and ≥20 DAP (SA20) and starchy endosperm at 12 DAP (SE12) and ≥20 DAP (SE20). LFQ intensities of proteins were averaged over three replications. Bars represent standard deviation. For statistical analyses we performed a Student's *t-*Test (*n* = 3). The *p*-values indicate significant quantitative changes of hordeins. **(C,D)** Immunofluorescence of hordeins with anti-gliadin antibody shows morphological changes of the hordein-positive PBs (indicated by arrows) between 12 DAP **(C)** and ≥20 DAP **(D)** in subaleurone (SA) and starchy endosperm (SE). Note the very faint signal in aleurone (AL) at ≥20 DAP. The first two panels show overview pictures of the bright field and the fluorescence channel, respectively. Scale bar = 20 μm. Asterisks indicate starch granule. **(E,F)** Fluorescent signal quantification of anti-gliadin. Bar plots of the fluorescent intensity of anti-gliadin in all tissues at 12 and ≥20 DAP and in aleurone, subaleurone, and starchy endosperm separately at 12 and ≥20 DAP. Areas (*n* = 10–13 for 12 DAP; *n* = 7–11 for ≥20 DAP) were quantified for aleurone (AL), subaleurone (SA), and starchy endosperm (SE) from at least 2 slides with two sections. For statistical analyses we performed a Student's *t-*test. Bars represent standard deviation. Note the indicated *p*-values.

Two of the most abundant proteins within the set of 10 proteins identified in our LMD proteomics study were D-hordein and B3-hordein (Table S2 sheet A). A detailed proteomic analysis of the spatio-temporal distribution of D-hordein and B3-hordein showed that D-hordein was significantly more abundant in starchy endosperm than in the aleurone at 12 DAP (Figure 3A). B3-hordein accumulated the most in starchy endosperm at 12 and ≥20 DAP (Figure 3B). Interestingly, no B3-hordein could be detected in subaleurone at ≥20 DAP.

Recently, we showed that an increase of the relative protein abundance of hordoindolines was accompanied by their re-localization from PBs to the protein matrix of starch granules (Shabrangy et al., 2018). Here, we used a polyclonal rabbit anti-wheat gliadin that has been developed to recognize the main hordein families (B-hordeins, C-hordeins, γ-1-hordein, γ−2-hordein, γ−3-hordein, D-hordein) (Møgelsvang and Simpson, 1998; Tanner et al., 2013; Hensel et al., 2015). Immunofluorescence studies of hordein confirmed a detection of hordeins in PBs in the subaleurone and in the starchy endosperm at 12 and ≥20 DAP (Møgelsvang and Simpson, 1998; Hensel et al., 2015; Figures 3C,D). At 12 DAP, hardly any signal could be observed in the aleurone, whereas a very faint signal could be detected in the aleurone at ≥20 DAP (Figures 3C,D). Hordeins accumulated at enlarged PBs in the subaleurone and at scattered PBs in the starchy endosperm at ≥20 DAP (Figures 3C,D). Additionally, signals could be observed at the protein matrix at the periphery of starch granules in the subaleurone and starchy endosperm at 12 and ≥20 DAP (Figures 3C,D). We quantified the fluorescence signal created by anti-gliadin in the aleurone, subaleurone, and starchy endosperm at 12 and ≥20 DAP and observed a significant difference of the signal between the aleurone and subaleurone/starchy endosperm at 12 DAP and between the aleurone and starchy endosperm at ≥20 DAP; the signal always was most abundant in starchy endosperm followed by the subaleurone (Figure 3E). In contrast, a significant difference of the fluorescence signal could only be observed in the aleurone layer compared to the subaleurone and the starchy endosperm, where the signal was stronger at ≥20 DAP (Figure 3F).

These analyses revealed that hordeins are most abundant in starchy endosperm and that their relative abundance increased significantly in starchy endosperm between 12 and ≥20 DAP. This increase of hordein accumulation in developing starchy endosperm was accompanied by an additional localization of hordeins at the protein matrix at the periphery of starch granules.

### HvPDIL1-1 identification and a dynamic spatio-temporal relative protein abundance and localization during barley endosperm development

As hordeins accumulated in ER-derived PBs, we assumed that ER structures should be more abundant in the starchy endosperm at later development stages. Thus, a semi-quantitative microscopic approach was performed to quantify ER abundance in the aleurone, subaleurone, and starchy endosperm at 6 and ≥20 DAP. Barley sections were stained with the fluorescent ER-Tracker™ (Green), which effectively and specifically visualizes ER in barley seeds (Ibl et al., 2014).

**Figure 4:**
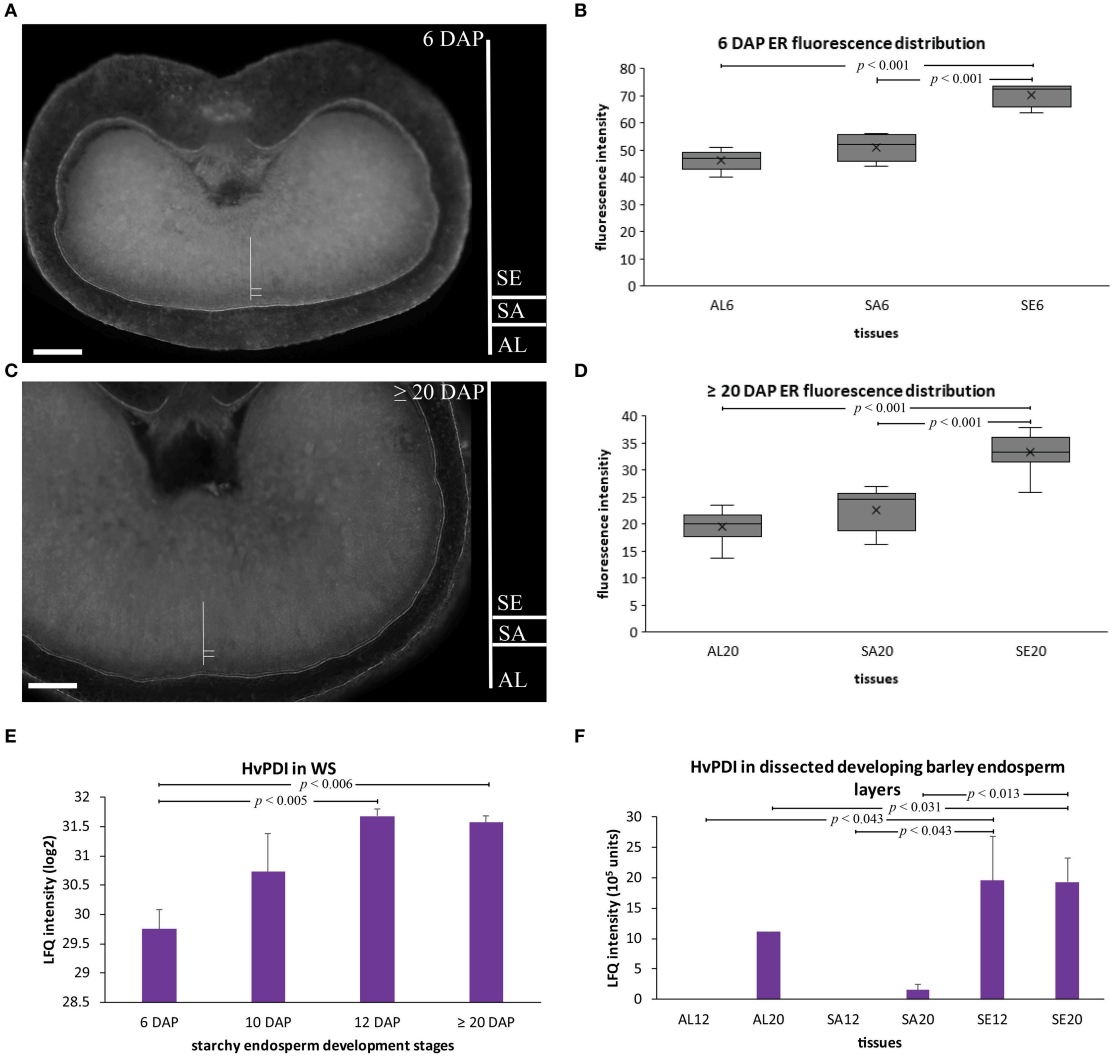
ER semi-quantitative fluorescence quantification and HvPDI relative protein abundance in developing barley endosperm. **(A–D)** Fluorescence quantification of the labeled ER membrane (using ER-Tracker™ Green) of sections prepared at 6 DAP **(A,B)** and ≥20 DAP **(C,D)**. The quantified areas of aleurone (AL), subaleurone (SA), and starchy endosperm (SE) are indicated. scale bar = 100 μm. Descriptive boxplot statistics of the fluorescence quantity show a significant increase in the ER abundance between aleurone (AL) and starchy endosperm (SE) between 6 to ≥20 DAP, respectively (*n* = 5 for 6 DAP, *n* = 10 for ≥20 DAP). AL6 = aleurone 6 DAP, SA6 = subaleurone 6 DAP, SE6 = starchy endosperm 6 DAP, AL12 = aleurone 12 DAP, AL20 = aleurone ≥20 DAP, SA12 = subaleurone 12 DAP, SA20 = subaleurone ≥20 DAP, SE12 = starchy endosperm 12 DAP, SE20 = starchy endosperm ≥20 DAP. **(E)** LFQ intensities of HvPDI in the whole seed (WS) at 6, 10, 12, and ≥20 DAP. HvPDI predominantly accumulated at mid- and late development stages of developing barley endosperm. LFQ intensities of proteins were averaged over three replications. Bars represent standard deviation. For statistical analyses we performed a Student's *t-*test (*n* = 3). The *p*-values are indicated. Note the log2 transformation of the protein abundance dataset of the WS. **(F)** LFQ intensities of HvPDI in aleurone (AL) at 12, ≥20 DAP and in starchy endosperm (SE) at 12, ≥20 DAP. The prominent HvPDI abundance in the starchy endosperm (SE) at 12 and ≥20 DAP. LFQ intensities of proteins were averaged over three biological replicates. Bars represent standard deviation. The *p-*values are indicated.

At 6 and ≥20 DAP, the strongest fluorescent signal could be observed in the starchy endosperm (Figures 4A–D), indicating the most abundant ER there.

Indeed, we could identify by our MS-based proteomics on the basis of 26 peptides a protein-disulfide isomerase (HvPDI) was among the strongest expressed proteins in aleurone, subaleurone and starchy endosperm (Figure S3, Table S2 sheet F). Additionally, our previous shotgun proteomics analysis of grain-filling processes identified and quantified HvPDI based on 44 unique matching peptides (Shabrangy et al., 2018) and revealed a continuous increase of HvPDI between 6 and 12 DAP that remained stable to ≥20 DAP (Figure 4E). Spatio-temporal proteomic analysis showed that HvPDI was most abundant in aleurone at ≥20 DAP and in starchy endosperm at 12 and ≥20 DAP (Figure 4F).

**Figure 5:**
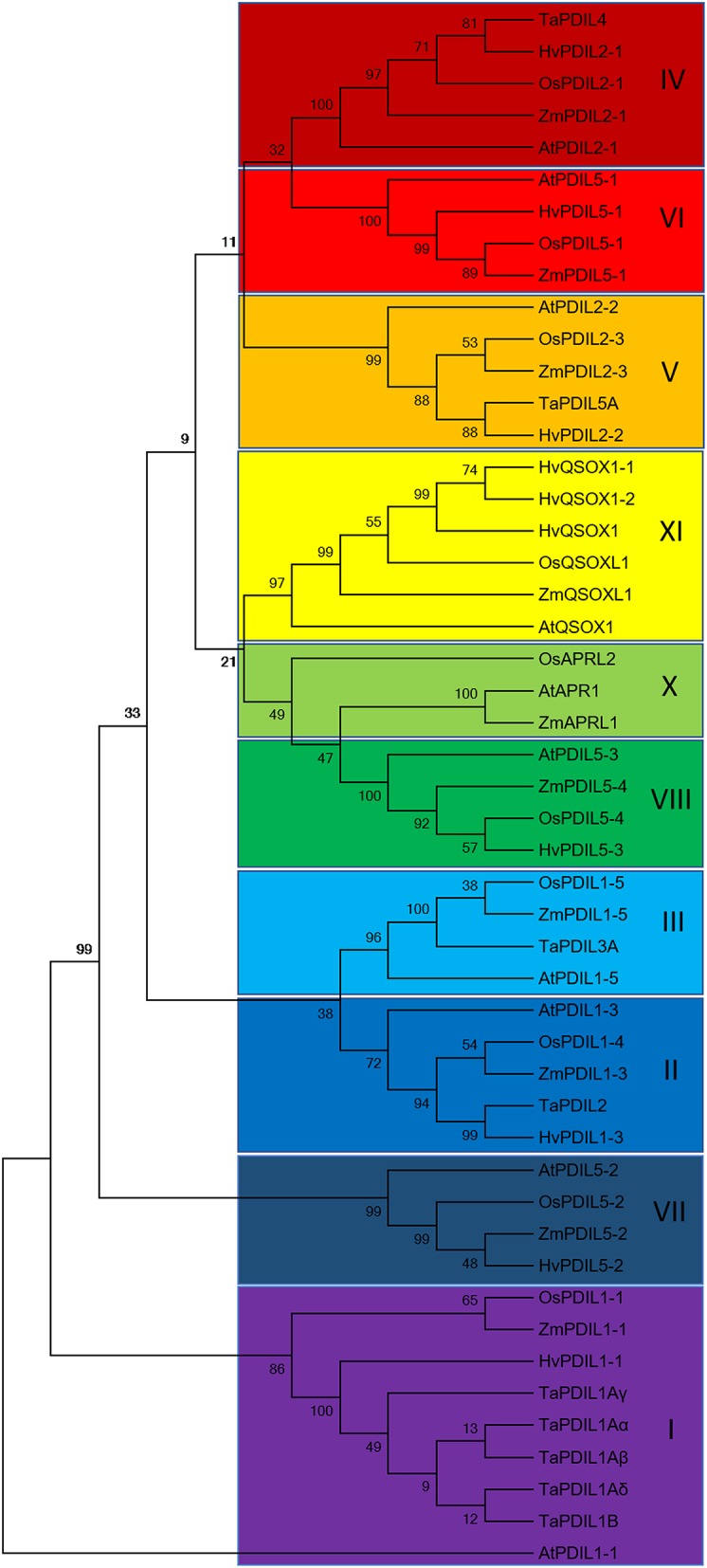
Rooted phylogenetic tree of *Arabidopsis (At), Oryza sativa ssp. japonica (Os), Zea mays (Zm), Triticum aestivum (Ta)* and *Hordeum vulgare (Hv)* PDI proteins resulting from neighbor-joining analysis of protein sequences using MEGA7.0.21 (Kumar et al., 2016). The 10 phylogenetic groups are indicated by the colored boxes. AtPDIL1-1 was used as an outgroup.

To further characterize the identified HvPDI protein, we used the known inventory of plant PDIs of Arabidopsis, rice (*O. sativa* ssp*. Japonica*), maize (*Z. mays*), and wheat (*T. aestivum*) to search for orthologous proteins in barley (Houston et al., 2005; Table 1, Figure S3). The phylogenetic analysis grouped HvPDI to cluster I as an ortholog of AtPDIL1-1, OsPDIL1-1, and TaPDI1 proteins (Figure 5, Figure S3). Thus, the identified PDI was termed HvPDIL1-1.

**Table 1:** Inventory of PDI members in *Arabidopsis* (At), *Oryza sativa* ssp*. japonica* (Os)*, Zea mays (Zm), Triticum aestivum (Ta)*, and *Hordeum vulgare (Hv)* classified into 10 phylogenetic groups.

**Group**	**Plant**	**PDIL protein**	**Gene name**	**cDNA**	**Protein—Uniprot**
I	Arabidopsis	AtPDIL1-1	At1g21750	NM_102024	Q9XI01
	*Oryza sativa* subsp. *Japonica*	OsPDIL1-1	Os11g0199200	AK068268	Q53LQ0
	*Zea mays*	ZmPDIL1-1	GRMZM2G091481	AY739284	Q5EUE1
	*Triticum aestivum*	TaPDIL1Aα	nd	AB933341	Q7FYS2
		TaPDIL1Aβ	nd	AB933345	A0A024FRZ0
		TaPDIL1Aγ	nd	AB933342	A0A024FSC1
		TaPDIL1Aδ	nd	AB933343	A0A024FR39
		TaPDIL1B	nd	AB933344	A0A024FRN3
	*Hordeum vulgare* subsp. *Vulgare*	HvPDIL1-1	NIASHv1066J19	AK357991	F2D284; A0A287NWJ5
II	Arabidopsis	AtPDIL1-3	At3g54960	NM_115353	Q8VX13
	*Oryza sativa* subsp. *Japonica*	OsPDIL1-4	Os02g0100100	AK071514	Q67IX6
	*Zea mays*	ZmPDIL1-3	GRMZM2G134889	AY739286	Q5EUD9
	*Triticum aestivum*	TaPDIL2	nd	AB933346	A0A024FRA5
	*Hordeum vulgare* subsp. *Vulgare*	HvPDIL1-3	NIASHv2105A13	AK370108	F2E1T7
III	Arabidopsis	AtPDIL1-5	At1g52260	NM_104105	A3KPF5
	*Oryza sativa* subsp. *Japonica*	OsPDIL1-5	Os06g0163400	AK073970	Q5WA72
	*Zea mays*	ZmPDIL1-5	GRMZM2G014076	AY739295	Q5EUD0
	*Triticum aestivum*	TaPDIL3A	nd	AB933347	D8L9A3
IV	Arabidopsis	AtPDIL2-1	At2g47470	NM_130315	O22263
	*Oryza sativa* subsp. *Japonica*	OsPDIL2-1	Os05g0156300	AK062024	Q75M08
	*Zea mays*	ZmPDIL2-1	GRMZM2G128171	AY739288	Q5EUD7
	*Triticum aestivum*	TaPDIL4D	nd	AB933348	D8L9B3
	*Hordeum vulgare* subsp. *Vulgare*	HvPDIL2-1	FLbaf131g21	AK249580	A0A287EWS7
V	Arabidopsis	AtPDIL2-2	At1g04980	NM_100376	Q9MAU6
	*Oryza sativa* subsp. *Japonica*	OsPDIL2-3	Os09g0451500	AK062254	Q67UF5
	*Zea mays*	ZmPDIL2-3	GRMZM2G389173	AY739290	Q5EUD5
	*Triticum aestivum*	TaPDIL5A	nd	AB933349	A0A024FRN8
	*Hordeum vulgare* subsp. *Vulgare*	HvPDIL2-2	NIASHv2020M08	AK364007	F2CPT7
VI	Arabidopsis	AtPDIL5-1	At1g07960	NM_202059	Q8GYD1
	*Oryza sativa* subsp. Japonica	OsPDIL5-1	Os03g0287900	AK063663	Q10N04
	*Zea mays*	ZmPDIL5-1	GRMZM2G073628; GRMZM2G443655	AY739291	Q5EUD4
	*Hordeum vulgare* subsp. *Vulgare*	HvPDIL5-1	FLbaf79k03	AK250421	A0A287P666
VII	Arabidopsis	AtPDIL5-2	At1g35620	NM_103262	Q94F09
	*Oryza sativa* subsp. *Japonica*	OsPDIL5-2	Os04g0432500	AK069367	Q0JD42
	*Zea mays*	ZmPDIL5-2	GRMZM2G113629	AY739292	Q5EUD3
	*Hordeum vulgare* subsp. *Vulgare*	HvPDIL5-2	FLbaf134g15	AK251979	M0WGB3
VIII	Arabidopsis	AtPDIL5-3	At3g20560	NM_112948	Q9LJU2
	*Oryza sativa* subsp. *Japonica*	OsPDIL5-4	Os07g0524100	AK099660	Q69SA9
	*Zea mays*	ZmPDIL5-4	GRMZM2G067063	AY739294	Q5EUD1
	*Hordeum vulgare* subsp. *Vulgare*	HvPDIL5-3	NIASHv1015H08	AK355066	F2CTW4
XI	Arabidopsis	AtQSOX1	At1g15020	AY062528	Q8W4J3
	*Oryza sativa Japonica* subsp.	OsQSOXL1	Os05g0552500	AK121660	Q6AUC6
	*Zea mays*	ZmQSOXL1	GRMZM2G113216	AY739305	Q5EUC0
	*Hordeum vulgare* subsp. *Vulgare*	HvQSOX1	NIASHv2001P04	AK362034	F2DDR7
		HvQSOX1-1	NIASHv1003K24	AK353910	F2CQK8
		HvQSOX1-2	NIASHv2100M17	AK369902	F2E181
X	Arabidopsis	AtAPR1	At4g04610	NM_116699	P92979
	*Oryza sativa* subsp. *Japonica*	OsAPRL2	Os06g0220800	AY739306	Q67VZ8
	*Zea mays*	ZmAPRL1	Apr	AY739296	Q5EUC9

**Figure 6:**
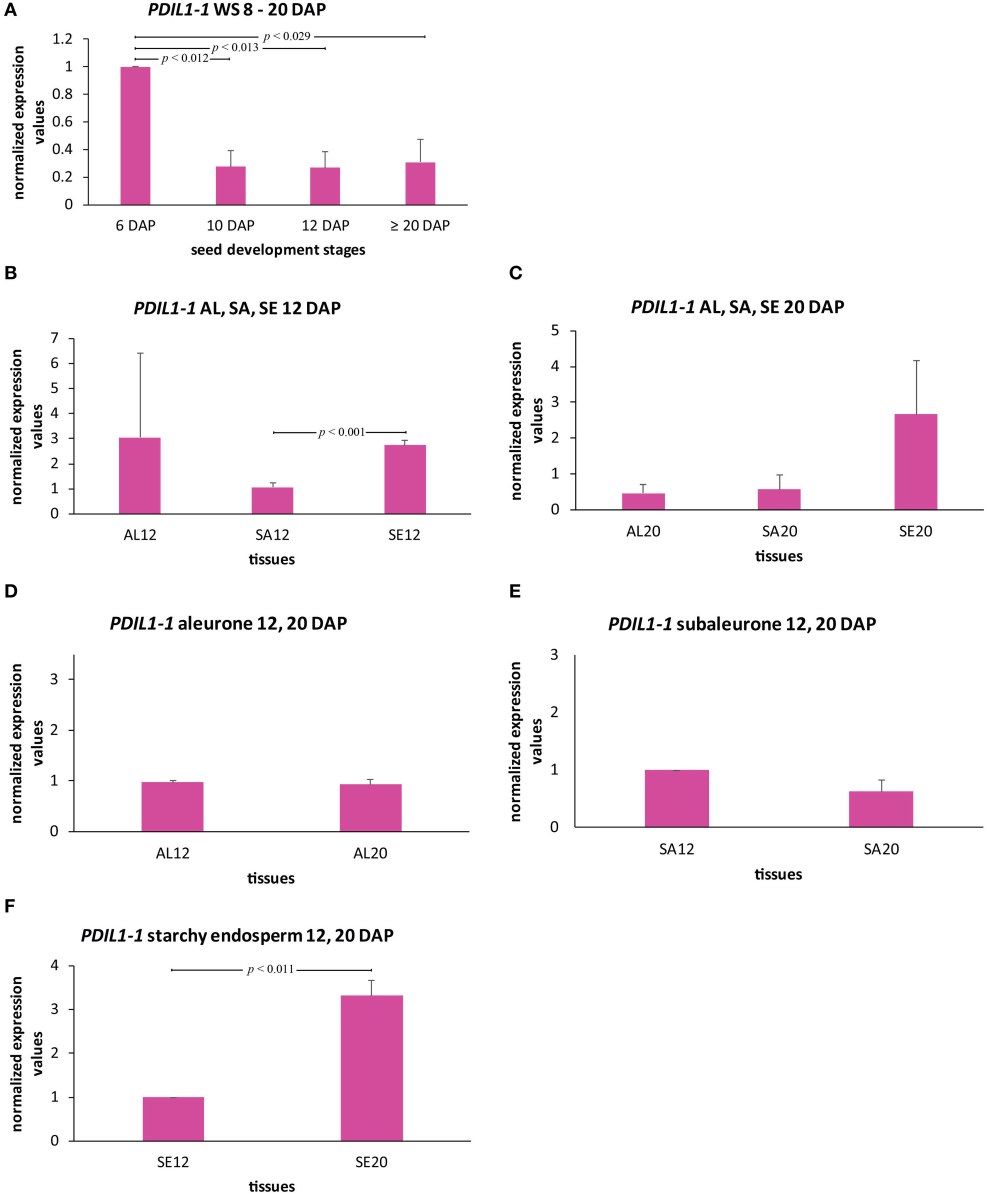
Spatio-temporal quantification of *HvPDIL1-1* transcripts in developing barley endosperm. **(A)** Bar graph describes the average over three biological replicates of the normalized transcripts from *HvPDIL1-1* in the whole seed (WS) at 6, 10, 12, and ≥20 DAP. **(B–F)** *HvPDIL1-1* transcript quantification in all tissues at 12 DAP **(B)**, at ≥20 DAP **(C)**, and in aleurone **(D)**, subaleurone (SA) **(E)**, and starchy endosperm **(F)** at 12 and 20 DAP. For statistical analyses we performed a Student's *t-*test (*n* = 3). Bars represent standard deviation. Note the indicated *p*-values.

To gain insight into to expression behavior of HvPDIL1-1, we analyzed the transcript level of HvPDIL1-1 during barley endosperm development by RT-qPCR (Figures 6A–F). RNA was isolated from whole seeds harvested at 6, 10, 12, and ≥20 DAP, and the previously characterized most stable reference genes were used to normalize the *HvPDIL1-1* transcripts as described in Shabrangy et al. (2018). The *HvPDIL1-1* transcript in the WS was most abundant at 6 DAP followed by a significant decrease at 10 DAP and remained stable to ≥20 DAP (Figure 6A). Northern-blot analysis of *OsPDI* mRNA in developing rice seeds showed an increase from 3 to 7 DAP, followed by a decline (Takemoto et al., 2002). Here, the *HvPDIL1-1* transcript abundance followed the same trend.

To further characterize the spatio-temporal transcript abundance of *HvPDIL1-1* during grain filling processes, LMD was used to dissect the aleurone, subaleurone, and starchy endosperm at 12 DAP and ≥20 DAP to perform RT-qPCR analysis using the previously described reference genes for normalization (Shabrangy et al., 2018). At 12 DAP, the *HvPDIL1-1* transcript was significantly more abundant in the starchy endosperm than the subaleurone (Figure 6B). At ≥20 DAP, the transcript amount of *HvPDIL1-1* was higher, but not significantly in the starchy endosperm than the aleurone and subaleurone (Figure 6C). RT-qPCR analyses of aleurone, subaleurone, and starchy endosperm identified significant differences of the *HvPDIL1-1* transcript in starchy endosperm between 12 and ≥20 DAP but not in aleurone and subaleurone (Figures 6D–F). Taken together, even though the *HvPDIL1-1* transcript was most abundant during early grain filling stages, spatio-temporal analyses revealed high levels of *HvPDIL1-1* transcripts in starchy endosperm at 12 and at ≥20 DAP, respectively.

**Figure 7:**
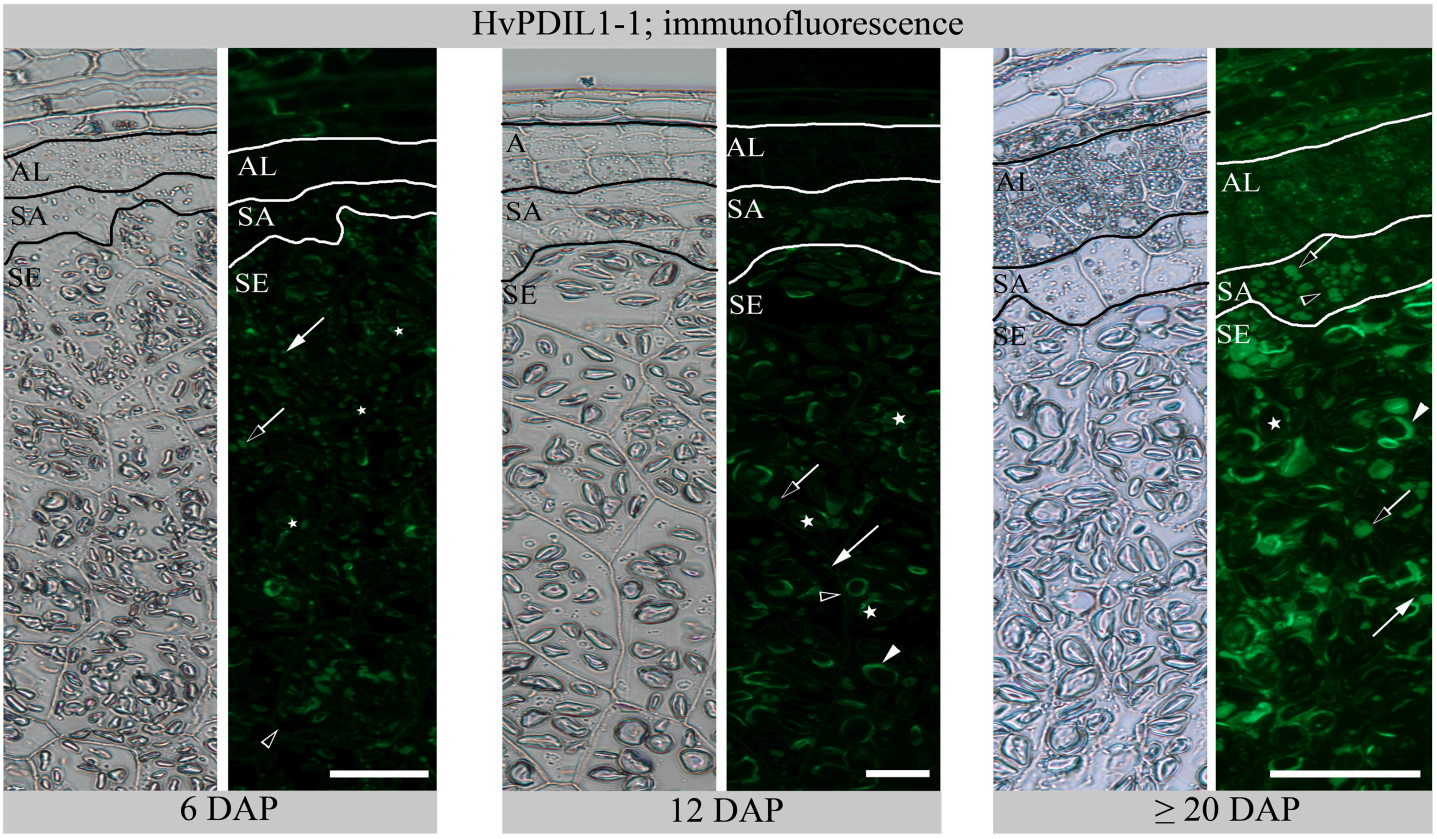
Immunofluorescence of HvPDIL1-1 with anti-AtPDIL1-1 at 6, 12, and ≥20 DAP. Aleurone (AL), subaleurone (SA), starchy endosperm (SE). Arrows indicate PBs with a smaller diameter, open arrows indicate PBs with a larger diameter and arrowheads the specific signal from the protein matrix at the periphery of the starch granules (asterisks). See the labeling of the plasma membrane in starchy endosperm (SE) at 12 DAP and in aleurone (AL) and subaleurone (SA) at ≥20 DAP (open arrowheads). Note the weak signal in aleurone (AL) at ≥20 DAP. Scale bar = 50 μm.

In order to explore the spatio-temporal subcellular distribution of HvPDIL1-1 during endosperm development, we prepared semi-thin sections of seeds harvested at 6, 12, and ≥20 DAP. We used anti-AtPDIL1-1 (kindly provided by Dr. Richard Strasser) that was previously described to recognize HvPDI-positive PBs in barley endosperm (Farid et al., 2011; Hensel et al., 2015). Whereas a weak signal appeared from PBs in subaleurone and starchy endosperm at 6 DAP, additional signal could be observed at the protein matrix at the periphery of starch granules in starchy endosperm at 12 DAP; at ≥20 DAP, the signal intensity from PBs and from the periphery of starch granules increased (Figure 7). We quantified the diameter of the anti-AtPDIL1-1-positive PBs in subaleurone and starchy endosperm at 6, 12, and ≥20 DAP where we could observe that the diameter of the anti-AtPDIL1-1-positive PBs significantly increased between 6 and ≥20 DAP in subaleurone, whereas in starchy endosperm the diameter of PBs significantly decreased between 6 and ≥20 DAP (Figure S4). This surprising outcome results from the diverging appearance of very small stand-alone and larger, fused PBs at 12 and at ≥20 DAP (Figure 7). As can also be seen, anti-AtPDIL1-1-labeled labeled structures were additionally detected in aleurone at ≥20 DAP. These results indicate the specific tissue dynamics of ER structures during barley endosperm development that are most prominent in starchy endosperm at ≥20 DAP.

### The developing starchy endosperm proteome

Our LMD proteomic data show a high accumulation of SSPs and HvPDIL1-1 proteins in the starchy endosperm, indicating a prominent role of the starchy endosperm in SSP accumulation. In this context, we decided to conduct a second proteomics analysis only on the starchy endosperm with a higher amount of material and including 6 DAP as an early development stage time-point. A total of 157 proteins were quantified, out of which 35 proteins changed significantly their relative protein abundance (Table S2 sheets C). Unsupervised multivariate statistics clustered the different starchy endosperm stages separately as indicated by principal component analysis (PCA) (Figure S6, Table S2 sheet E). PCA analysis revealed that cluster PC1 accounted for 62.8% of the total variance, while PC2 accounted for 10.9% of the variance.

To further understand which metabolic processes are underlying the differences between 12 and ≥20 DAP, we investigated protein functions from PC1 negative loadings and PC2 positive loadings. The 10 lowest loadings on the PC1 mainly accounted for SSPs (hordein, gliadin, serpin related proteins), hordoindoline, alpha-amylase inhibitors (IAM1 and IAT3 homologs), and finally a beta-amylase related to starch degradation. Altogether, those proteins present a common accumulation trend from 6 to ≥20 DAP. Proteins with the 10 highest loadings on PC2 mainly accounted for the amino acid biosynthetic process, protein turnover (ribosomal proteins, eukaryotic initiation factors, ubiquitin-related proteins), thioredoxin, development (TCTP), and the SSPs gliadin and hordoindoline b1. Unlike the PC1 proteins, the PC2 proteins did not have a unified pattern, as did PC1, but 60% of them (amino-acid biosynthesis, protein translation, development, and thioredoxin) followed the same pattern as HvPDIL1-1.

**Figure 8:**
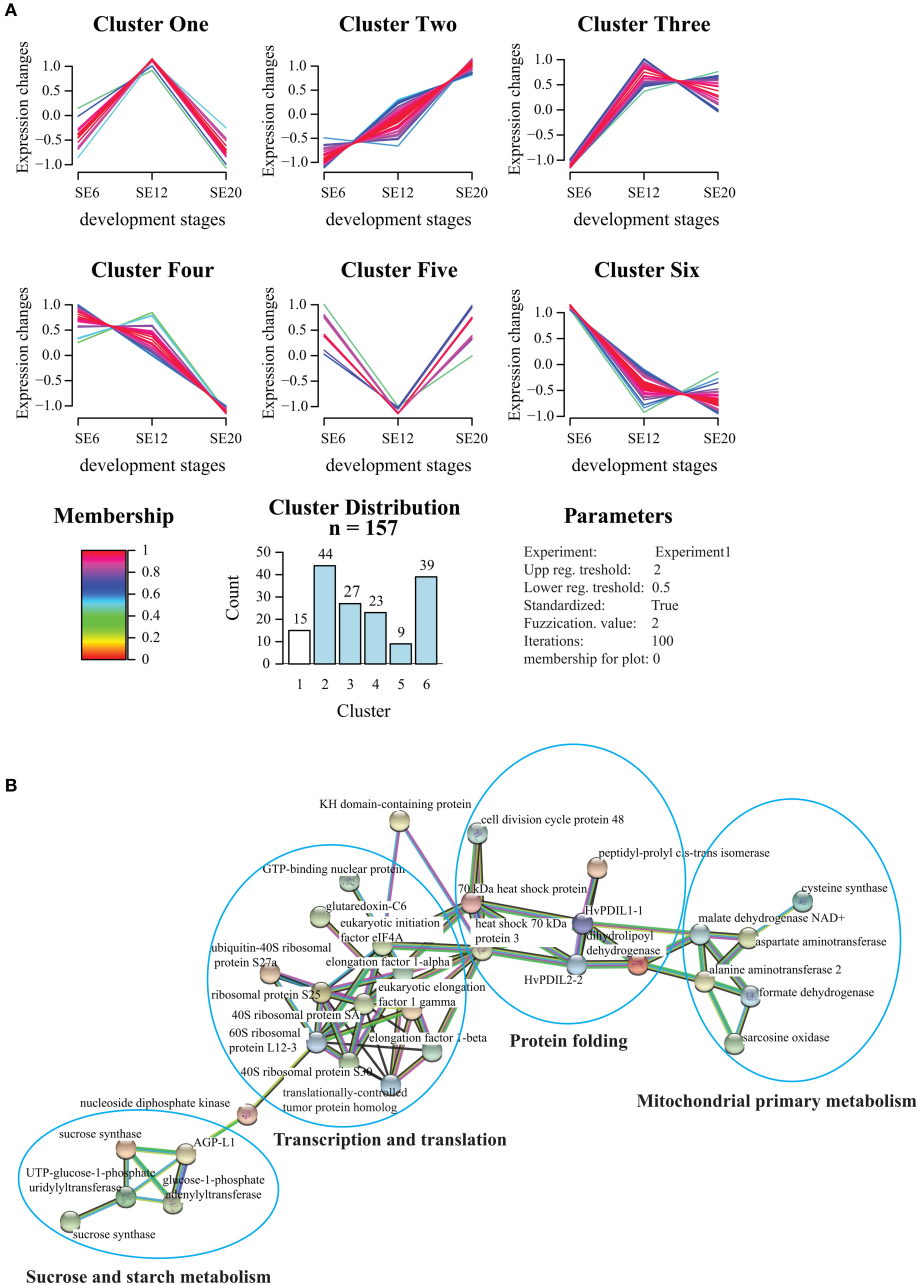
Functional analysis of the starchy endosperm proteome. **(A)** The 157 proteins detected and quantified by LC-MS out of the starchy endosperm LMD approach were clustered with the fuzzy algorithm from GproX (Rigbolt et al., 2011). In total 6 relevant clusters were identified (Table S2 sheet G). Membership value represents how well the protein profile fit the average cluster profile. Membership of plot = 0 indicate that no threshold was applied to plot protein abundance pattern. **(B)** Proteins with similar abundance patterns as HvPDIL1-1 present in cluster Three (and some in cluster Four) were analyzed by STRING database. STRING default parameters were used (Franceschini et al., 2013). Protein names are indicated.

GProX software (Rigbolt et al., 2011) was used for unsupervised clustering to partition the temporal profiles of the 157 proteins into six clusters (Figure 8A). The clusters revealed stage-specific relative protein abundance pattern of many functionally related proteins occurring at distinct time points. Interestingly, Cluster One as well as few proteins in Cluster Four revealed relative protein abundance patterns closely related to HvPDIL1-1 in Cluster Three. To understand the relationship between these proteins, protein-protein interaction analysis using the STRING database was conducted (Figure 8B). Interestingly, proteins present in Clusters One and Three are part of four main interconnected functions: protein translation and its regulation, amino acid biosynthesis, protein folding and sucrose and starch metabolism. In contrast, 15 proteins belonging to Cluster Five, and to a lesser extent to Clusters Six and Two, present inverse trends compared to the HvPDIL1-1 relative protein abundance pattern. Among them, a grain softness protein-1 (M0VAF1), which is a key component of the hardness locus, is particularly interesting (Morris et al., 2013). Two other groups of Clusters showed opposite trends. The first group included Clusters Four and Six showing a decreasing relative protein relative abundance pattern over time, while Cluster Two showed a continuous increase. Cluster Two is essentially composed of SSPs and enzyme inhibitors, although some of the proteins are related to primary metabolism and defense mechanisms. In contrast, Clusters Four and Six present proteins related to translation, protein folding and chaperone as well as RNA binding functions. Other proteins related to degradation processes, redox homeostasis and hexose metabolism could also be detected (Figure S7, Table S2).

### The dynamic relative protein abundance behavior of HvPDIL1-1 correlates with ER rearrangement in the starchy endosperm

**Figure 9:**
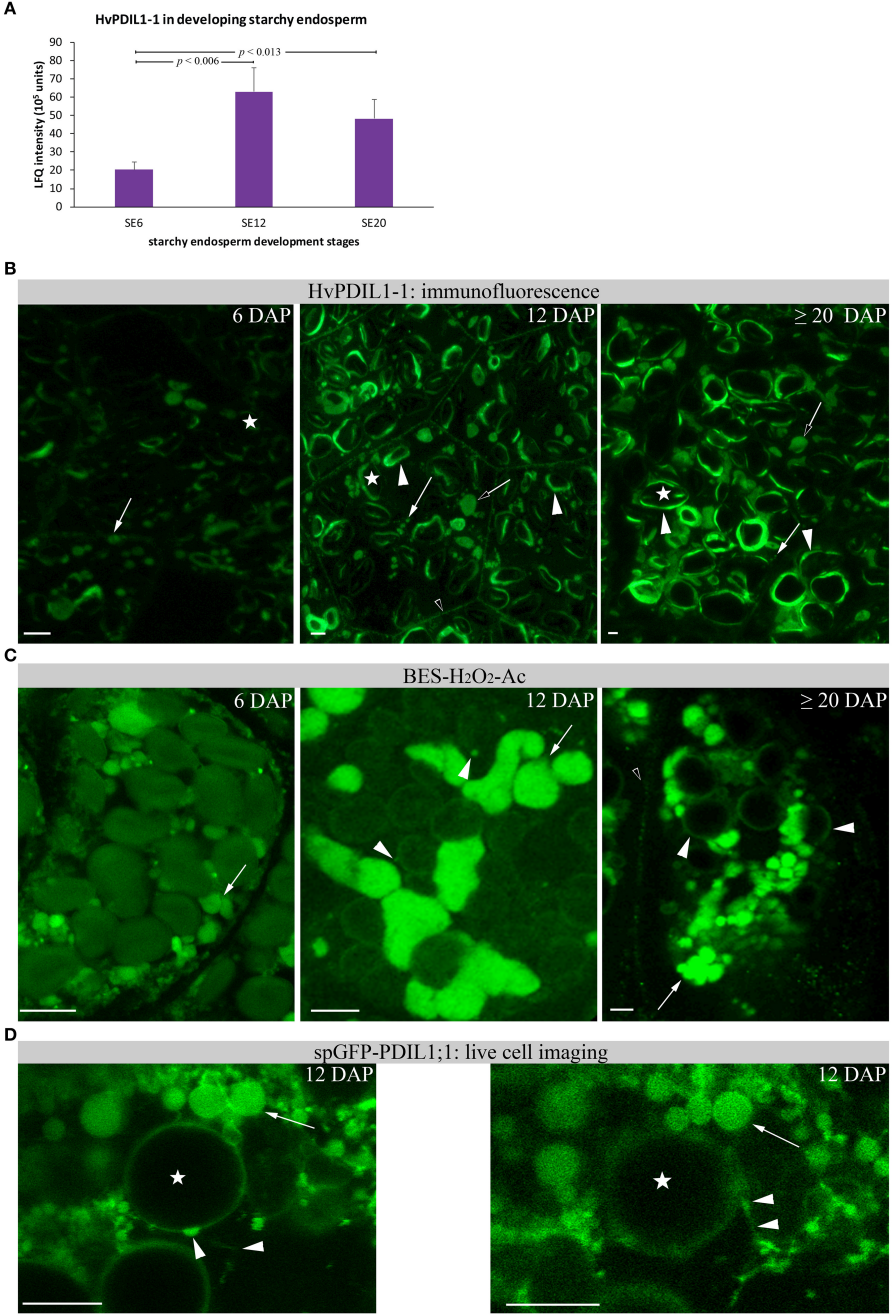
Relative HvPDIL1-1 protein abundance and localization changes in developing starchy endosperm (SE). **(A)** LFQ intensities of HvPDIL1-1 in starchy endosperm (SE) at 6 DAP (SE6), 12 DAP (SE12), and ≥20 DAP (SE20). LFQ intensities of proteins were averaged over three biological replicates. Bars represent standard deviation. For statistical analyses we performed a Student's *t-*test (*n* = 3). The *p*-values are indicated. Note the significant changes between SE6 and SE12 and SE20, respectively. **(B)** Immunofluorescence of HvPDIL1-1 with antiAt-PDIL1-1. Signals appear from PBs (arrow) that decrease their size between 12 and ≥20 DAP (Figure S4). Arrows indicate PBs with a smaller diameter, open arrows indicate PBs with larger diameter. The signal at the protein matrix at the periphery of starch granules was first recognized at 12 DAP (arrowhead) and increased at ≥20 DAP. Note the signal at the plasma membrane in SE at 12 DAP (open arrowhead). **(C)** BES-H_2_O_2_-Ac-positive PBs (arrows) were detected at 6 DAP and changed their morphology between 12 DAP and ≥20 DAP. Note the signal (vesicular structures) at the periphery of the starch granules (arrowhead). At ≥20 DAP, the signal around the starch granules increased. **(D)** Live cell imaging of spGFP-PDIL1;1 revealed spGFP-PDIL1;1-positive structures fusing with the protein matrix at the periphery of starch granules. Note the two images showing the fusion events within the movie. Images were acquired every 5.2 s. Asterisks indicate starch granules. Bar scale = 10 μm in all part figures. The corresponding movie is provided as Supplementary Material (Movie S1).

Given that the highest protein abundance of HvPDIL1-1 is in the starchy endosperm (Table S2), we wanted to follow the abundance and localization of HvPDIL1-1 specifically in the starchy endosperm during endosperm development (Table S2 sheet C). Using shotgun proteomics, we first quantified the appearance of D-hordein and B3-hordein, found in Cluster Two, in starchy endosperm at 6, 12, and ≥20 DAP. The abundance of both hordeins increased in starchy endosperm between 6 and ≥20 DAP (Figures S8A,B). In addition, immunofluorescence studies of hordeins revealed developmental changes. Whereas at 6 DAP the signal appeared predominantly in PBs (Figure S8C), hordeins were also localized at the protein matrix at the periphery of starch granules at 12 and ≥20 DAP (Figures S8D,E). These observations indicate possible fusion events of PBs with the protein matrix at the periphery of starch granules. We quantified the diameter of the anti-gliadin-positive PBs in starchy endosperm at 6, 12, and ≥20 DAP where we could observe that the diameter of PBs are significantly decreasing between 6 and ≥20 DAP in starchy endosperm (Figure S5). This result correlates with the reduced sizes of anti-AtPDIL1-1-positive PBs in the starchy endosperm at ≥20 DAP (Figure S4). Next, we analyzed the relative protein abundance of HvPDIL1-1 in starchy endosperm at 6, 12, and ≥20 DAP. HvPDIL1-1 was predominantly detected in starchy endosperm at 12 and ≥20 DAP, showing a significant increase between 6 and 12 DAP and 6 and ≥20 DAP (Figure 9A). No significant difference could be observed between the relative protein abundance of HvPDIL1-1 at 12 and ≥20 DAP (Figure 9A). The increase in the relative protein abundance of HvPDIL1-1 was accompanied by a re-localization of HvPDIL1-1 in developing starchy endosperm: although the signal at 6 DAP was weak and appeared predominantly at small PBs, AtPDIL1-1 recognized HvPDIL1-1 at the protein matrix at the periphery of starch granules at 12 DAP that increased at ≥20 DAP (Figure 9B). Additionally, HvPDIL1-1 accumulated at the plasma membrane in the starchy endosperm at 6 and 12 DAP (Figure 9B).

In rice, spGFP-OsPDIL1;1 labeled PBs accumulate the fluorescence probe BES-H_2_O_2_-Ac, indicating H_2_O_2_ production in rough ER (Onda et al., 2009). BES-H_2_O_2_-Ac was therefore used to determine whether H_2_O_2_ was generated in starchy endosperm in spGFP-OsPDIL1;1–labeled PBs. Confocal microscopic analysis of starchy endosperm cells at 12 DAP showed positive PBs of different size and small dots labeled by BES-H_2_O_2_-Ac at the periphery of starch granules (Figure S9). As shown in Figure 9C, BES-H_2_O_2_-Ac labeled small PBs at 6 DAP that increased their volume at 12 DAP. At ≥20 DAP, strong BES-H_2_O_2_-Ac signals could be detected at small PBs and at the protein matrix at the periphery of starch granules (Figure 9C). The proximity of BES-H_2_O_2_-Ac-labeled PBs to the starch granules point to some putative fusion events (Figure 9C).

We generated a transgenic barley line with OsTIP3::spGFP-OsPDIL1;1 to analyze *in vivo* the ER dynamics of the developing starchy endosperm. OsTIP3::spGFP-OsPDIL1;1 has been used as an ER lumen marker in the rice SA layer to identify rough ER structure (Onda et al., 2009). Transgenic barley lines were created by the bombardment of immature embryos with particles coated in the corresponding expression construct. The presence of the transgene in the regenerated barley plants was confirmed by PCR. The full-length fusion construct was checked by Western blot (Figure S1). A time series for spGFP-PDIL1;1–labeled ER was taken where we could observe fusion events between dilated ER structures and dots at the periphery of the starch, indicating that spGFP-PDIL1;1 finally fused with the protein matrix at the periphery of starch granules (Figure 9D).

## Discussion

### Label-free proteome quantification of dissected aleurone, subaleurone, and starchy endosperm

The present study aimed to unravel the spatio-temporal proteome regulation in barley aleurone, subaleurone, and starchy endosperm by a multi-disciplinary strategy involving LC-MS analysis, microscopy, and transcript quantification. Experiment One aimed to analyze the spatio-temporal proteome dynamics in aleurone, subaleurone and starchy endosperm at 12 and ≥20 DAP (Figure 1A). As described, the LC-MS method used in Experiments One and Two could separate and identify peptides in a similar range as shown in previously published plant LMD data (Schad et al., 2005; Ramsay et al., 2006; Dembinsky et al., 2007; Kaspar et al., 2010). Because the aleurone and subaleurone hold less tissue area compared to the starchy endosperm, the total extracted protein amount was lower, even though the relative protein amount (μg/1,000,000 μm^2^) of samples only varied between distinct tissues and timepoints (Figure 1B). Interestingly, the diameter of the PBs increased significantly in subaleurone between 12 and ≥ 20 DAP (Figure S4A), thus may have an influence of the increased relative protein abundance in this tissue. Additionally, tissue heterogeneity between the aleurone, subaleurone and starchy endosperm was shown. Consequently, Experiment One presented a heterogeneous protein distribution result between the aleurone, subaleurone, and starchy endosperm.

As indicated by Experiment Two, a significant improvement could be achieved using higher protein amounts (Figure 1C). Thus, we could confirm the relation between peptide identification rate and protein content reported for mammals (Wang et al., 2005). The number of identified proteins also depends on the extraction buffer: (Mahalingam, 2017) previously mentioned the use of an extraction buffer containing Tris–HCl and KCl that was not successful in extracting SSPs such as hordeins, but favored the identification of lower abundance protein. Further analysis of dissected aleurone, subaleurone, and starchy endosperm layers at distinct time-points extracted by different buffers optimized for the extraction of tissue-specific cereal proteins may identify additional low-abundance proteins.

### The relative protein abundance and the localization of hordeins is spatio-temporally regulated in developing barley endosperm

PBs are special ER-derived cereal seed storage organelles in which hordeins, barley SSPs, are accumulated. *In vivo* microscopy together with electron microscopic analyses of distinct tissues of developing barley endosperm revealed a dynamic behavior of PBs during barley endosperm development: PBs appeared in the subaleurone and in the starchy endosperm at early development stages, finally fusing to, taken up and released by the protein storage vacuole (Cameron-Mills, 1980; Ibl et al., 2014). Our recent proteomic analysis of endosperm development revealed an increase of SSPs between 6 and ≥20 DAP (Shabrangy et al., 2018). Here, D-hordein and B-hordein could be detected in the proteome of dissected aleurone, subaleurone and starchy endosperm at 12 and ≥20 DAP (Figure 2). Both proteins showed an increase of abundance in the aleurone as well as the starchy endosperm between 12 and ≥20 DAP. Interestingly, the abundance of both proteins slightly decreased in the subaleurone between 12 and ≥20 DAP (Figures 3A,B). We used anti-gliadin antibody that identifies the main hordein family members, including B-hordein, C-hordein, γ-1-hordein, γ-2-hordein, γ-3-hordein, and D-hordein, to compare the subcellular distribution of hordeins with the quantification data.

Interestingly, we could localize hordeins in bulk PBs in the subaleurone layer (Figures 3C,D). The quantified hordeins in subaleurone at ≥20 DAP may already be at the lowest detection level for proteomics using LC-MS—especially considering the used extraction buffer - whereas in the immunofluorescence study, the main hordein family was detected. Besides the hordein quantification, microscopic analyses show that the hordein-labeled PBs appeared roundish in the starchy endosperm at 12 DAP but changed into more dispersed structures at ≥20 DAP (Figures 3C,D). Taken together, these results show a strong increase of D-hordeins and B3-hordeins in the starchy endosperm between 12 and ≥20 DAP, accompanied by morphological changes of hordein-positive PBs. It will be interesting to study the influence of abiotic stress on the spatio-temporal regulation of the main hordein family and on the barley food end-product quality, as it is known that abiotic stress strongly affects the GPC (Ashraf, 2014; Halford et al., 2015).

### ER structures and HvPDIL1-1 are most abundant in starchy endosperm in late development

As hordeins were deposited within ER-derived PBs and accumulated preferentially in starchy endosperm at 12 and ≥20 DAP, we assumed that ER structures as well as ER-related proteins or proteins involved in the generation of PBs would be most abundant in the starchy endosperm. Indeed, our microscopic semi-quantitative analysis of ER fluorescence in aleurone, subaleurone, and starchy endosperm at 6 and ≥20 DAP indicated that ER is most abundant in starchy endosperm (Figures 4A–D). In line with the ER fluorescence analysis, LC-MS and RT-qPCR analyses of HvPDI provided valuable insights into the spatio-temporal distribution of HvPDI during barley endosperm development. Using shotgun proteomics approach of the whole barley seed we observed that HvPDI increased between 6 and 12 DAP and decreased at ≥20 DAP (Figure 4E). Our proteomic analyses of aleurone, subaleurone, and starchy endosperm at 12 and ≥20 DAP revealed a preferential accumulation of HvPDI in starchy endosperm, especially at 12 DAP, which remained stable at ≥20 DAP (Figure 4F). Our results are consistent with previous proteomic studies in developing barley grains, as HvPDIs continuously increased until 10–29 DAP (Møgelsvang and Simpson, 1998; Kaspar-Schoenefeld et al., 2016). Additionally, bioinformatic analyses identified HvPDI as an ortholog of AtPDIL1-1 (Figure 5). This agrees with the expression levels of wheat TaPDIL1 orthologs in developing caryopses were highest between 10 and 15 DAP and among TaPDI family proteins (Kimura et al., 2015). The transcript level of *HvPDIL1-1* did not correlate with the relative protein abundance of HvPDIL1-1 in whole seed as well as in spatio-temporal analyses (Figure 6). This high protein abundance, low mRNA abundance discrepancy has already been recognized and discussed for maize endosperm (Walley et al., 2013). Walley et al. suggested three possible scenarios that could explain the poor correlation between transcript and protein levels including diurnal transcript level cycle, unstable mRNA and differences between the tissues where transcription and translation occur (Woo et al., 2001; Walley et al., 2013). Further experiments are necessary to consider in detail the discrepancy between transcript and protein abundances in developing barley endosperm, especially in distinct tissues at different time points. In this context, RNA localization studies are under way by our group to assess where HvPDIL1-1 mRNA is stored, accumulated, transported or degraded.

Additionally, immunolocalization of HvPDIL1-1 with anti-AtPDIL1-1 showed strong signals in the PBs and especially in the protein matrix at the periphery of starch granules in the starchy endosperm at ≥20 DAP (Figure 7). Hordeins as well as HvPDIL1-1 were close to the detection limit in the aleurone layer at 6 DAP. However, a careful analysis of the microscopic data revealed a plasma membrane localization of HvPDIL1-1 in the aleurone layer, indicating an additional subcellular function of HvPDIL1-1 at the plasma membrane or a different regulation of HvPDIL1-1 localization compared to hordeins. Altogether, our data are in line with rice and wheat results and suggest that ER structures, as well as HvPDIL1-1, are predominantly active in starchy endosperm (Kim et al., 2012; Kimura et al., 2015).

### The starchy endosperm proteome during development

Experiment Two was conducted to study the proteome of the dissected starchy endosperm specifically during development. Thus, we increased the amount of sample and included an additional early seed development timepoint, 6 DAP. As the high accumulation of hordeins and HvPDIL1-1 in the starchy endosperm indicated a role of this tissue in the SSP accumulation, we were especially interested to characterize these proteins. Cluster analyses revealed that HvPDIL1-1 clustered into Cluster Three, where most of the proteins first show an increase to 12 DAP followed by a decrease at ≥20 DAP (Figure 8A, Table S2 sheet G). Interestingly, 5 out of the 27 proteins are involved in redox processes (Table S2 sheet G). Proteins related to sucrose and starch metabolism, protein translation, and folding functions, followed a pattern similar to HvPDIL1-1 (Figure 8B). Those results suggest coordination between translation and energy metabolism. In addition, hordoindoline b1 was identified in Cluster Three (Table S2 sheet G). Hordoindolines (HINs), HINa, HINb1, and HINb2, are orthologs of wheat puroindolines (PINs), which are small, basic, cysteine-rich seed-specific proteins, and responsible for grain hardness (Darlington et al., 2001; Takahashi et al., 2010). Very recently we characterized HINs in developing barley endosperm and found the highest protein level of HINs in the starchy endosperm at ≥20 DAP, accumulated first in PBs and finally deposited at the periphery of starch granules (Shabrangy et al., 2018). D-hordein and B3-hordein clustered in the Cluster Two, where the proteins in the starchy endosperm show an increase of the relative protein abundance between 6 and ≥20 DAP (Table S2 sheet G). Most of the Cluster Two related proteins are involved in SSP related functions. Thus, ER-related proteins including HvPDIL1-1, SSPs, and proteins involved in oxidative protein folding may contribute in a significant manner to starchy endosperm activity at the mid- and late-development stages. Consequently, these proteins are possible targets for genetic manipulation of barley starchy endosperm in the future.

### The increase of the relative protein abundance of HvPDIL1-1 in the starchy endosperm is accompanied by its re-localization from PBs to the protein matrix at the periphery of starch granules

**Figure 10:**
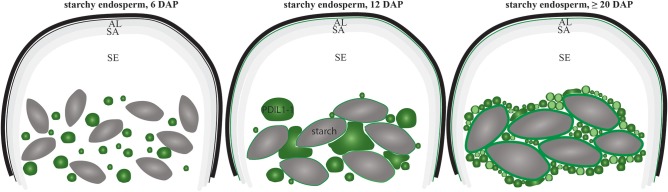
Schematic overview of the re-localization of HvPDIL1-1-positive PBs during starchy endosperm development. Note the decrease of the diameter of the HvPDIL1-1 labeled PBs (green) between 6 DAP and ≥20 DAP (summarizing Figure 7, Figures S4, S8). First fusing events with the protein matrix at the periphery of starch granules (gray structures) was observed at 12 DAP and continued at ≥20 DAP. AL, aleurone; SA, subaleurone; SE, starchy endosperm; Note the indicated starch granules. The localization of HvPDIL1-1 at the plasma membrane in the SE at 12 and ≥20 DAP is indicated as the plasma membrane is green colored (summarizing Figures 7, 9B).

Our proteomic quantification analyses showed that D-hordein, B-hordein, and HvPDIL1-1 are among the most abundant proteins in starchy endosperm (Table S2 sheets A,C). Specifically, the relative protein abundance of D-hordein, as well as B-hordein continuously increased in starchy endosperm between 12 and at ≥20 DAP. Interestingly, HvPDIL1-1 was most abundant at 12 DAP and remained stable to ≥20 DAP (Figure 9A), subsequently clustering in a different group than the hordeins in the proteomic analyses. However, both immunofluorescence studies of hordeins and HvPDIL1-1 identified a rearrangement from labeled PBs at 6 DAP to an additional signal localized in the protein matrix at the periphery of starch granules at ≥20 DAP (Figure 9B). Our microscopic studies further revealed localization of HvPDIL1-1 at the plasma membrane. Even though PDIs have been observed at plasma membranes before, the function behind this localization is still unknown (Kimura et al., 2015). In rat exocrine pancreatic cells, PDI was reported to be exported from the ER to the plasma membrane (Yoshimori et al., 1990). Posttranslational modifications or an overflow of the ER was suggested to be responsible for this localization. Here, we could detect spatio-temporal regulation of the localization of HvPDIL1-1 at the plasma membrane, as we observed this phenomenon in all tissues at 6, 12 DAP and only in the aleurone and subaleurone at ≥20 DAP (Figure 9B). The absence of HvPDIL1-1 from the plasma membrane in starchy endosperm at late development stages suggests that the plasma membrane in these cells is probably affected if the tissue is undergoing some programmed cell death. Additionally, BES-H_2_O_2_-Ac was used to detect H_2_O_2_ in PBs. Our microscopic results show the development-dependent changes of BES-H_2_O_2_-Ac-labeled PBs that were accumulating at the periphery of the starch granules, possibly followed by a fusion event to the protein matrix at the periphery of the starch granules (Figure 9C). Finally, live cell imaging allowed us to follow fusing events of spGFP-PDIL1;1-positve ER structures to the protein matrix around the starch granules (Figure 9D). In summary, our data indicate that the increase of HvPDIL1-1 in starchy endosperm during development was accompanied by its partial re-localization from PBs to the starch matrix at the periphery of starch granules (summarized in Figure 10).

### Possible roles of HvPDIL1-1 in starchy endosperm development

Besides their nutritional importance, barley seeds have been used to produce recombinant proteins. PBs have an “inert” character and are characterized by a protective matrix and have therefore been as the favorite organelle for targeting and storing recombinant proteins (summarized in Tschofen et al., 2016). We found HvPDIL1-1 most highly expressed in the starchy endosperm across all studied time points. Additionally, we could detect HvPDIL1-1 in PBs and in the protein matrix at the periphery of starch granules. This information could be useful for industrial applications. For instance, the specialized seed storage organelles, the ER-derived PBs, give recombinant proteins a protective environment that is especially important for molecular farming products (Hofbauer and Stoger, 2013). The recombinant proteins in transgenic cereal seeds remain stable and active for several years (Doran, 2006; Boothe et al., 2010). In this context, protein targeting plays a crucial role in achieving higher yields. Certainly, proteins targeted to specific subcellular compartments are more stable and protected from degradation (Martoglio and Dobberstein, 1998; Hofbauer and Stoger, 2013). An >10-fold increase in recombinant seed protein levels relative to cytoplasmic relative abundance was observed due to the presence of chaperone proteins as PDI and an oxidizing environment (Greenham and Altosaar, 2013). Barley seed-derived products include growth factors, cytokines and epithelial growth factor in barley seeds (summarized in Tschofen et al., 2016). Further experiments are necessary to determine the enzymatic properties of HvPDIL1-1 and to confirm a strong oxidative refolding activity for HvPDIL1-1 in barley. Wheat PDI ortholog proteins that are categorized as group I PDI family proteins exhibit the most oxidative refolding activities of all wheat PDIs. In rice, spGFP-OsPDIL1;1-derived signals appeared from rough ER and rough ER–derived PBs that accumulated BES-H_2_O_2_-Ac, which indicates the generation of H_2_O_2_ in the rough ER and in the PBs (Onda et al., 2009). Therefore, it is legitimate to speculate that HvPDIL1-1 may share this high activity (Kimura et al., 2015). This is of special interest concerning the production of recombinant proteins in PBs and the effect of HvPDIL1-1 on their stability.

In line with modulation of the HvPDIL1-1expression, another example associated with a downregulation of HvPDIL1-1 could be found for bread industry. Indeed, PDI family proteins in wheat were detected at the protein matrix and in wheat flour, and their oxidative refolding activity was inhibited by bacitracin (Koh et al., 2010; Kimura et al., 2015). A possible effect on the food product quality has been seen, as TaPDIL1 was noted as playing a role in retaining glutenin macro polymers during dough mixing (Koh et al., 2010; Kimura et al., 2015). Rice flour prepared from *esp2* seeds, which lacks OsPDIL1;1, showed superior bread-making qualities compared to wild-type flour. Successful tests obtained from *esp2* seeds led to the speculation that formed protein complexes, including esp2 SSPs, improve the extensibility and plasticity of the dough (Onda et al., 2009). Thus, a spatio-temporal barley starch granule proteome may reveal new insights into the granule-bound proteins that are attached to the surface and thus contribute to the properties and qualities of the starch for various food and non-food applications.

## Conclusion

LMD enables the study of the proteome of dissected aleurone, subaleurone, and starchy endosperm at different developmental time-points making it very useful in the study of spatio-temporal regulated proteins in the heterogeneous tissue of barley endosperm. Here, we provide data that identifies the starchy endosperm as the most prominent storage tissue for SSPs accompanied by a high abundance of ER structures and the accumulation of HvPDIL1-1. Hordeins as well as HvPDIL1-1 are specifically deposited in the starchy endosperm and re-localize subcellularly from PBs to the protein matrix at the periphery of starch granules. Further experiments are necessary to decipher how HvPDIL1-1 affects the properties of starch granules and cereal SSPs. Finally, electron microscopy will be used to unravel the trafficking route of HvPDIL1-1 to its distinct subcellular localization. Indeed, it was discussed that the extent of protein synthesis has an effect of the final deposition of hordeins in ER and that in rice and wheat PDIL1-1 is important for the correct deposition of SSPs. Specific downregulation of HvPDIL1-1 by, for example CRISP/Cas9, will help to understand the impact of HvPDIL1-1 on the formation of SSP in barley. Further, a possible effect of HvPDIL1-1 on food end-product properties and on plant molecular farming products should be investigated.

## Author contributions

VR, SR, and VI designed the experiments. VR, SR, VI, P-JR, MW, EK, and AS conducted the experiments and analyzed data. ES and WW discussed data and results. VR and VI wrote the manuscript.

### Conflict of interest statement

The authors declare that the research was conducted in the absence of any commercial or financial relationships that could be construed as a potential conflict of interest.

## Supplementary material

Table S1 *HvPDIL1-1* is described by its gene product function, cDNA (UniProt) entry, primer sequence, and amplicon length.

Table S2 LMD MS data of Experiment One and Experiment Two. Sheet 1: index: letter = sheet; sheet A = Protein group quantified in experiment 1; sheet B = Identified peptides corresponding to the identified protein group from experiment 1; sheet C = Protein group quantified in experiment 2; sheet D = Identified peptides corresponding to the quantified protein group from experiment 2; sheet E = Loading of the PCA for experiment 2; sheet F = Peptides identified and quantified for HvPDIL1-1 in experiment 1; sheet G = List of protein of the experiment 2 associated to identified clusters calculated by GProX (see section Materials and Methods); Corresponding protein names and String identifiers are indicated accordingly to Figure 8B.

Movie S1 A time series (4 s interval, 2 min movie) for spGFP-PDIL1;1 labeled ER was taken by confocal microscopy. Note the fusion events of the GFP labeled ER to the periphery of the starch granule.

Figure S1 Western blot of spGFP-PDIL1;1 transgenic barley line. Anti-GFP was used to detect GFP-PDIL1;1 with the corresponding molecular weight of 80 kDa. Note the intact fusion protein and that no signal could be detected in the negative control (GP).

Figure S2 cDNA alignment of HvPDIs. cDNA alignment performed by MEGA7.0.21 (Kumar et al., 2016) and visualized by GeneDoc (Nicholas and Nicholas, 1997). The conserved percentage is shown as following: black = 100%, dark gray = 80%. Primers are indicated in bold.

Figure S3 Protein alignment of plant PDIs of Arabidopsis (At), rice (*Oryza sativa* subsp*. Japonica*, Os), maize (*Zea mays*, Zm), and wheat (*Triticum aestivum*, Ta). Alignment was performed by MEGA7.0.21 (Kumar et al., 2016) and visualized by GeneDoc (Nicholas and Nicholas, 1997). The conserved percentage is shown as following: black = 100%, dark gray = 80%. Peptide hits are indicated in bold.

Figure S4 Diameter size of anti-AtPDIL1-1 labeled PBs in subaleurone and starchy endosperm during development. **(A)** Diameter (μm) of the PBs increased significantly in subaleurone between 6 and ≥20 DAP. **(B)** Diameter (μm) of the PBs decreases significantly in starchy endosperm between 6 and ≥20 DAP. The diameter of 100 PBs in both endosperm layer (subaleurone and starchy endosperm) was measured at 6, 12, and ≥20 DAP, respectively.

Figure S5 Diameter size of anti-gliadin-labeled PBs in starchy endosperm during development. Diameter (μm) of the PBs decreases significantly in starchy endosperm between 6 and ≥20 DAP. The diameter of 100 PBs in and starchy endosperm was measured at 6, 12, and ≥20 DAP, respectively.

Figure S6 PCA was conducted on logarithmically transformed relative protein abundance **(A)**, each dot corresponds to a single replication (*n* = 3). Protein loadings, on PC1 and PC2 were projected in the two-dimensional plan. **(B)** The 10 lowest loadings on PC 1 and the 10 highest loadings on PC 2 are colored in orange and blue, respectively. More detail on those proteins is provided in Table S2 sheet E, showing loadings of EX2, and in the main text.

Figure S7 Functional analysis of the starchy endosperm proteome. Proteins identified in Clusters Four and Six (Figure 8, Table S2sheetG) were analyzed with STRING database. STRING default parameters were used (Franceschini et al., 2013).

Figure S8 The relative protein abundance of D-hordein, B hordein and their distribution in starchy endosperm. **(A)** LFQ intensities of D-hordein significantly increase between 6 and ≥20 DAP in the starchy endosperm (SE). **(B)** B-hordein is significantly increasing between 6 and 12 DAP and 6 and ≥20 DAP in the starchy endosperm (SE). SE6 = starchy endosperm 6 DAP, SE12 = starchy endosperm 12 DAP, SE20 = starchy endosperm ≥20 DAP. LFQ intensities of proteins were averaged over three replications. Bars represent standard deviation. For statistical analyses we performed a Student's *t-*test (*n* = 3). The *p-*values are indicated. **(C–E)** Immunofluorescence analyses of B-hordein with anti-gliadin. Note the distribution change of B-hordein: at 6 DAP **(C)**, anti-gliadin labels predominantly PBs (arrow) at 12 DAP **(D)** and ≥20 DAP **(E)**, the signal also appears at the protein matrix at the periphery at starch granules (arrowheads). Asterisks indicate starch granules. Scale bar = 20 μm.

Figure S9 Colocalization of PBs, BES-H_2_O_2_-Ac, and ER-Tracker™ Red. Starchy endosperm section of 12 DAP was stained with BES-H_2_O_2_-Ac and ER-Tracker™ Red and analyzed by confocal microscopy. PBs were visualized by autofluorescence (asterisk). Colocalization could be observed with the green fluorescence appearing by BES-H_2_O_2_-Ac-labeled PB (arrowhead). Note the ER membrane around the PB (arrow). Scale bar = 5 μm.

## Abbreviations

ACPIII: acyl carrier protein III
AL: aleurone
At: Arabidopsis
ARF: ADP-ribosylation factor
DAP: days after pollination
ELF: elongation factor 1-alpha
ER: endoplasmic reticulum
ERO1: ER oxidoreductase 1
FBPA: fructosebisphosphatealdolase
GAP: glyceraldehyde-3-phosphate dehydrogenase
GPC: grain protein content
GRP: glycine-rich protein
HIN: hordoindoline
HSP70: Heat shock 70 kD protein
Hv: *Hordeum vulgare*
HSP90: heat shock protein 90
LMD: laser microdissection
Os: *Oryza sativa* ssp. japonica
PCA: principal component analysis
PDI: protein disulfide isomerase
PB: protein body
SA: subaleurone
SAM: S'adenosyl-L-methionine
SE: starchy endosperm
SSP: seed storage protein
SPE: solid phase extraction
Ta, *Triticum aestivum*, TRX: thioredoxin
UBI: ubiquitin gene
WS: whole seed
Zm: *Zea mays*.
