# Supplementary figures and images for: Microscopic and Proteomic Analysis of Dissected Developing Barley Endosperm Layers Reveals the Starchy Endosperm as Prominent Storage Tissue for ER-Derived Hordeins Alongside the Accumulation of Barley Protein Disulfide Isomerase (HvPDIL1-1)
Source: Front Plant Sci. 2018 Sep 10;9:1248. doi: 10.3389/fpls.2018.01248 (PMC6139375; doi:10.3389/fpls.2018.01248)

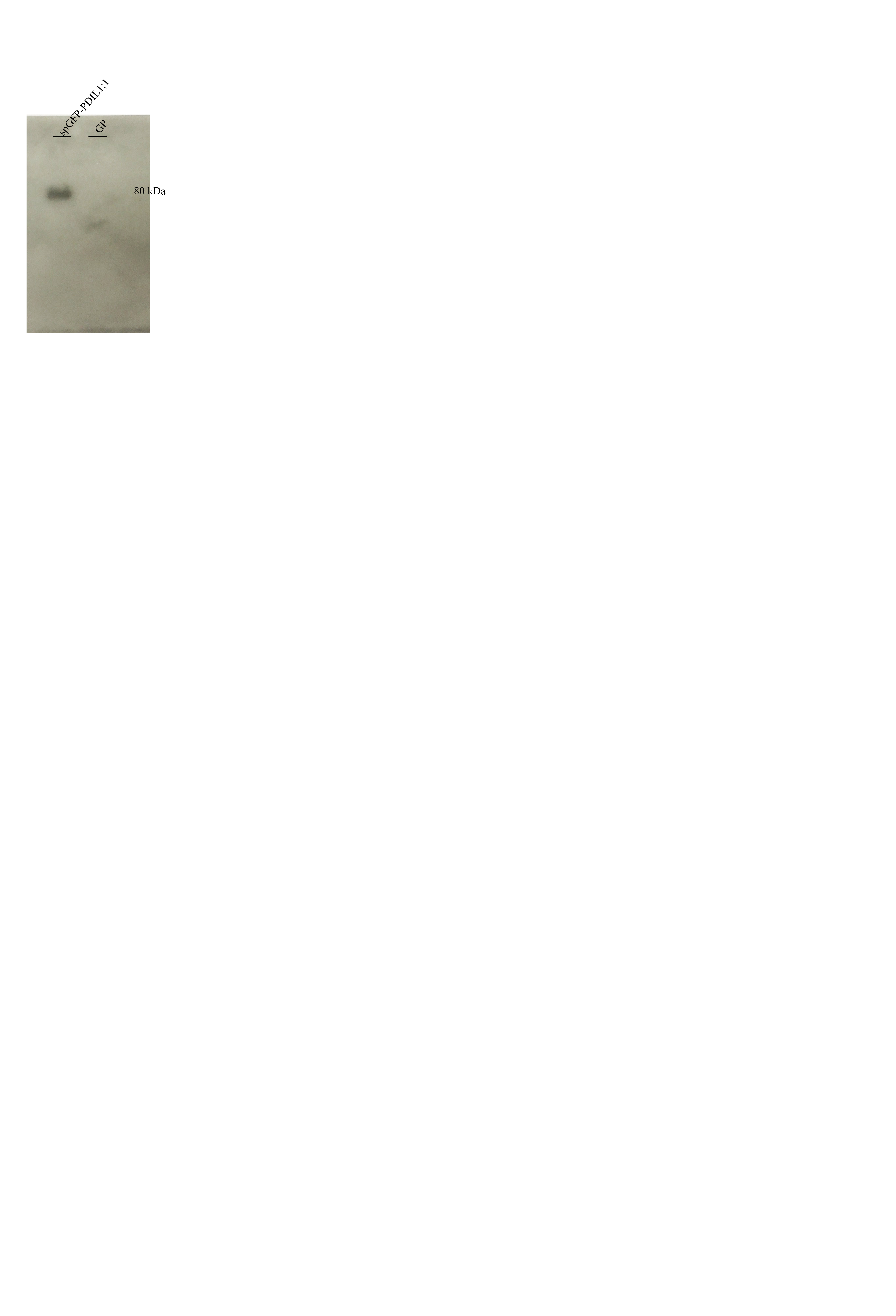

Supplement: Figure S1 — Western blot of spGFP-PDIL1;1 transgenic barley line. Anti-GFP was used to detect GFP-PDIL1;1 with the corresponding molecular weight of 80 kDa. Note the intact fusion protein and that no signal could be detected in the negative control (GP). [file Image_1.tif]

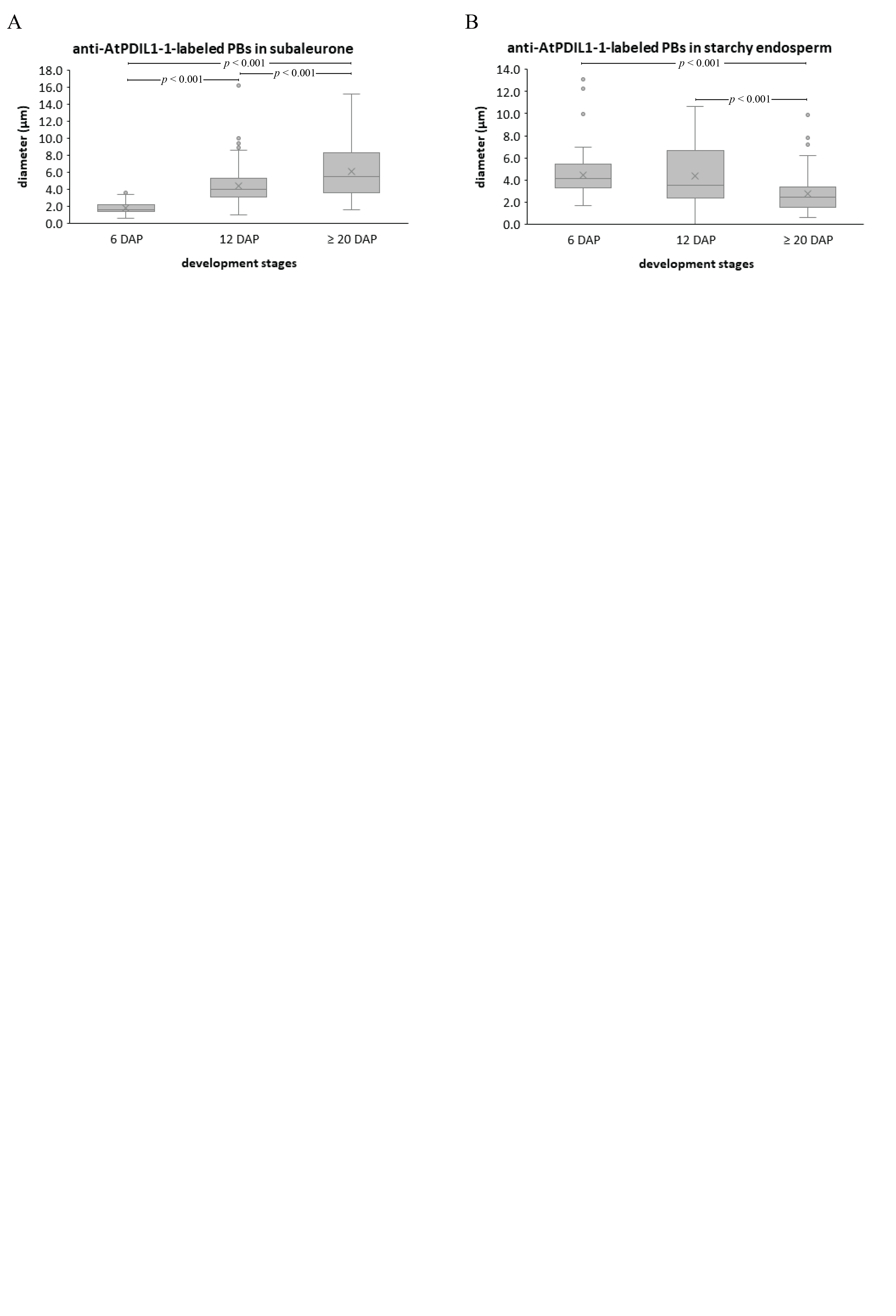

Supplement: Figure S4 — Diameter size of anti-AtPDIL1-1 labeled PBs in subaleurone and starchy endosperm during development. (A) Diameter (μm) of the PBs increased significantly in subaleurone between 6 and ≥20 DAP. (B) Diameter (μm) of the PBs decreases significantly in starchy endosperm between 6 and ≥20 DAP. The diameter of 100 PBs in both endosperm layer (subaleurone and starchy endosperm) was measured at 6, 12, and ≥20 DAP, respectively. [file Image_4.TIF]

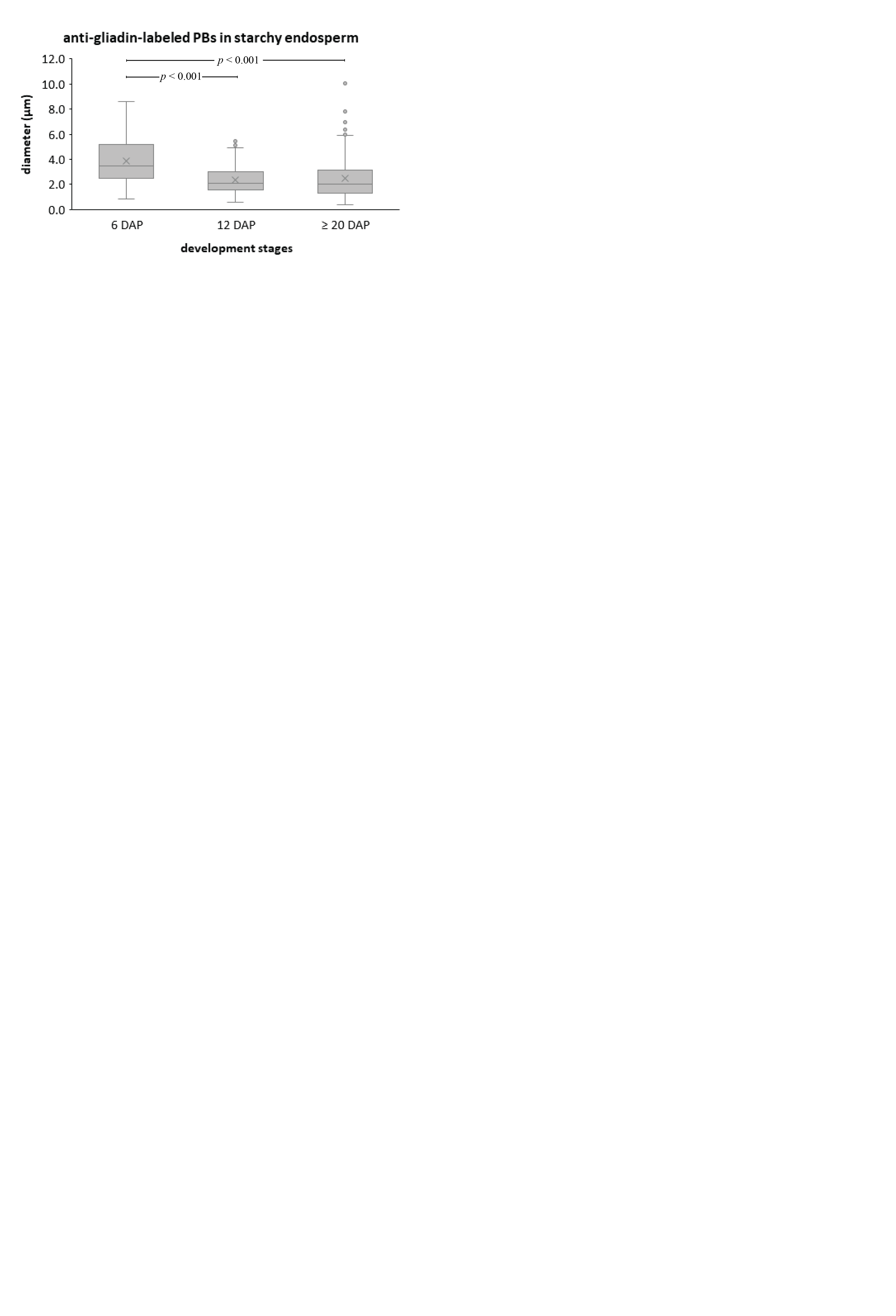

Supplement: Figure S5 — Diameter size of anti-gliadin-labeled PBs in starchy endosperm during development. Diameter (μm) of the PBs decreases significantly in starchy endosperm between 6 and ≥20 DAP. The diameter of 100 PBs in and starchy endosperm was measured at 6, 12, and ≥20 DAP, respectively. [file Image_5.TIF]

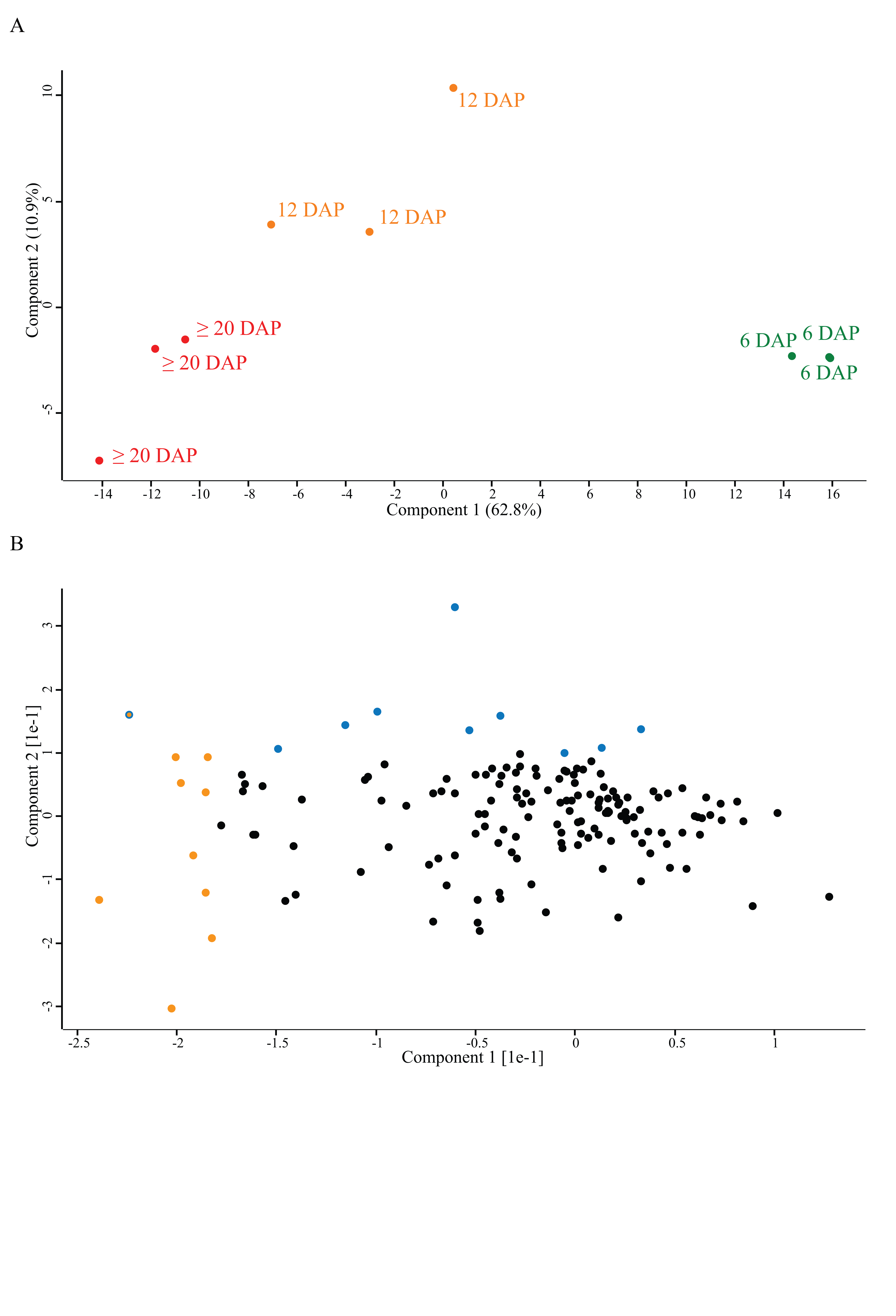

Supplement: Figure S6 — PCA was conducted on logarithmically transformed relative protein abundance (A), each dot corresponds to a single replication (n = 3). Protein loadings, on PC1 and PC2 were projected in the two-dimensional plan. (B) The 10 lowest loadings on PC 1 and the 10 highest loadings on PC 2 are colored in orange and blue, respectively. More detail on those proteins is provided in Table S2 sheet E, showing loadings of EX2, and in the main text. [file Image_6.TIF]

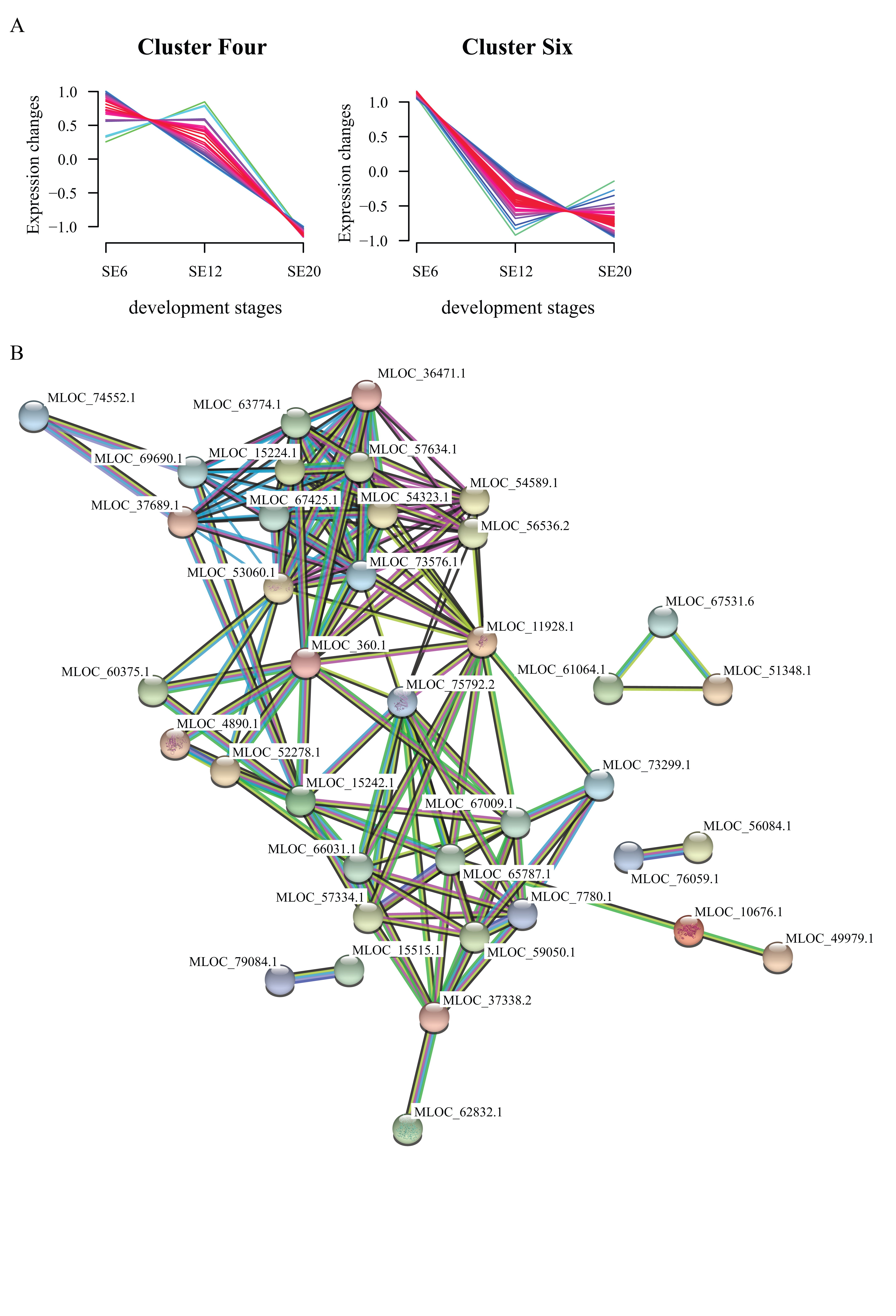

Supplement: Figure S7 — Functional analysis of the starchy endosperm proteome. Proteins identified in Clusters Four and Six (Figure 8, Table S2sheetG) were analyzed with STRING database. STRING default parameters were used (Franceschini et al., 2013). [file Image_7.TIF]

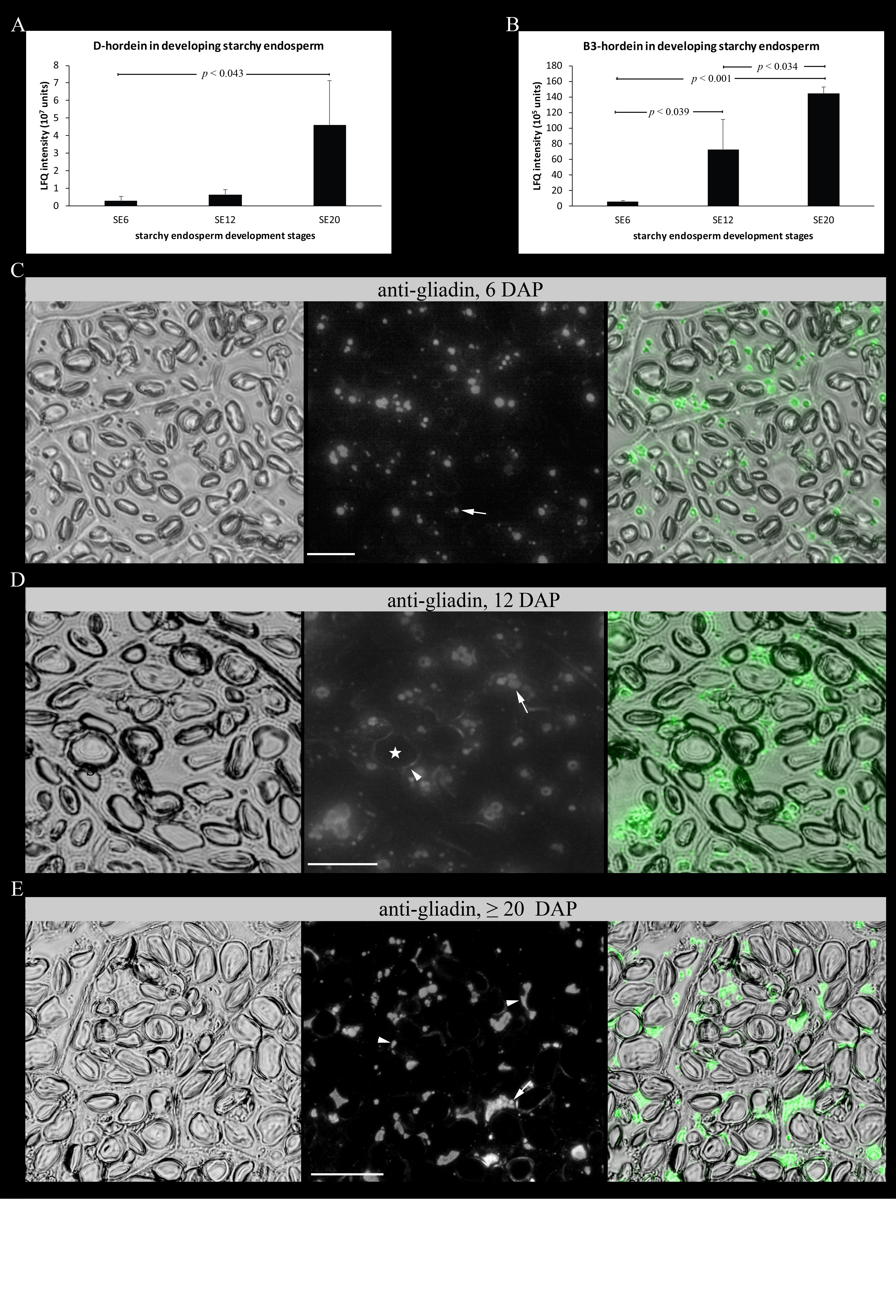

Supplement: Figure S8 — The relative protein abundance of D-hordein, B hordein and their distribution in starchy endosperm. (A) LFQ intensities of D-hordein significantly increase between 6 and ≥20 DAP in the starchy endosperm (SE). (B) B-hordein is significantly increasing between 6 and 12 DAP and 6 and ≥20 DAP in the starchy endosperm (SE). SE6 = starchy endosperm 6 DAP, SE12 = starchy endosperm 12 DAP, SE20 = starchy endosperm ≥20 DAP. LFQ intensities of proteins were averaged over three replications. Bars represent standard deviation. For statistical analyses we performed a Student's t-test (n = 3). The p-values are indicated. (C–E) Immunofluorescence analyses of B-hordein with anti-gliadin. Note the distribution change of B-hordein: at 6 DAP (C), anti-gliadin labels predominantly PBs (arrow) at 12 DAP (D) and ≥20 DAP (E), the signal also appears at the protein matrix at the periphery at starch granules (arrowheads). Asterisks indicate starch granules. Scale bar = 20 μm. [file Image_8.JPEG]

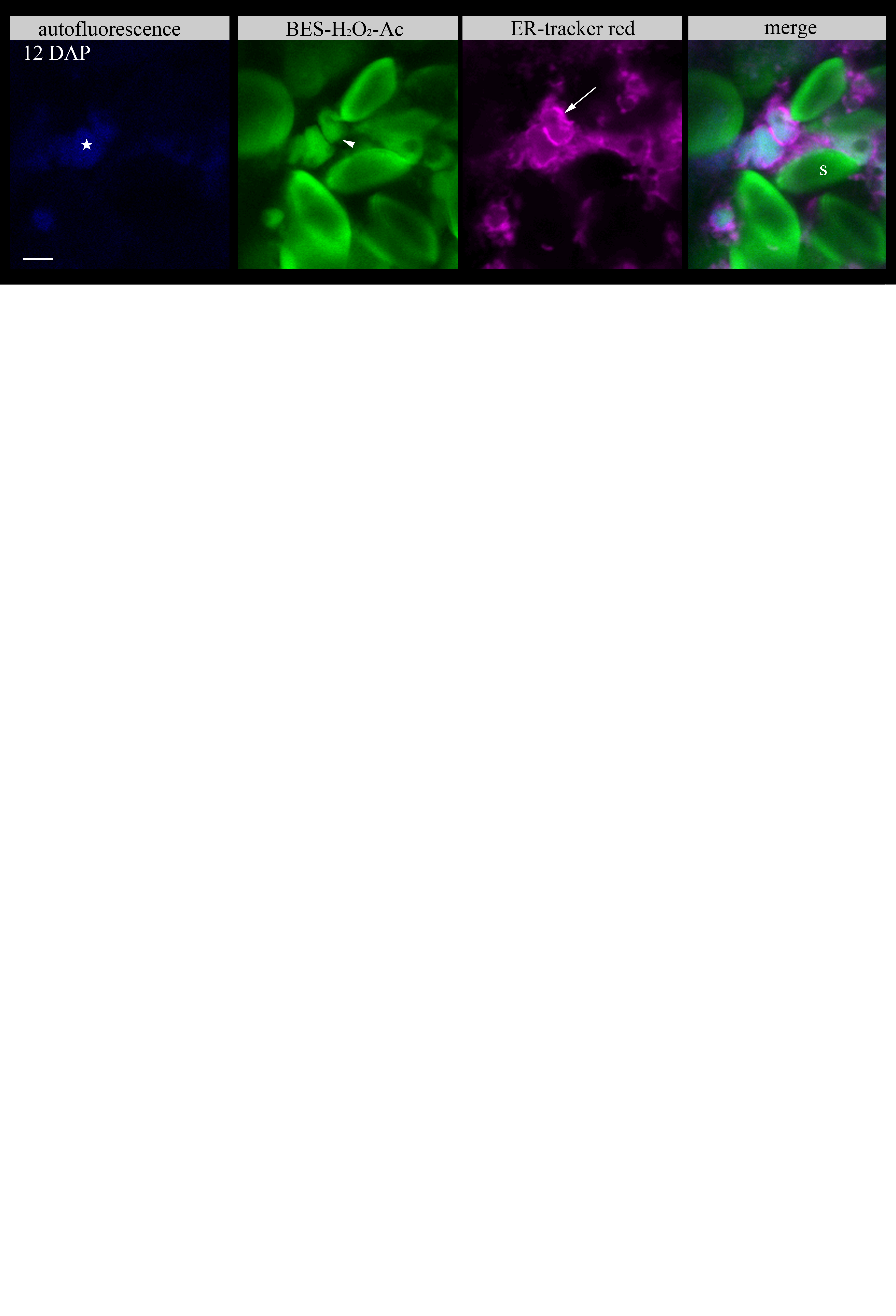

Supplement: Figure S9 — Colocalization of PBs, BES-H2O2-Ac, and ER-Tracker™ Red. Starchy endosperm section of 12 DAP was stained with BES-H2O2-Ac and ER-Tracker™ Red and analyzed by confocal microscopy. PBs were visualized by autofluorescence (asterisk). Colocalization could be observed with the green fluorescence appearing by BES-H2O2-Ac-labeled PB (arrowhead). Note the ER membrane around the PB (arrow). Scale bar = 5 μm. [file Image_9.TIF]
